# Opsin 1 and Opsin 2 of the Corn Smut Fungus *Ustilago maydis* Are Green Light-Driven Proton Pumps

**DOI:** 10.3389/fmicb.2019.00735

**Published:** 2019-04-10

**Authors:** Sabine Panzer, Annika Brych, Alfred Batschauer, Ulrich Terpitz

**Affiliations:** ^1^Theodor-Boveri-Institute, Department of Biotechnology and Biophysics, Biocenter, Julius Maximilian University, Würzburg, Germany; ^2^Department of Plant Physiology and Photobiology, Faculty of Biology, Philipps University, Marburg, Germany

**Keywords:** *Ustilago maydis*, patch-clamp, fungal rhodopsins, microbial rhodopsins, acetate, indole-3-acetic acid, structured illumination microscopy, sporidia

## Abstract

In fungi, green light is absorbed by rhodopsins, opsin proteins carrying a retinal molecule as chromophore. The basidiomycete *Ustilago maydis*, a fungal pathogen that infects corn plants, encodes three putative photoactive opsins, called *ops1* (UMAG_02629), *ops2* (UMAG_00371), and *ops3* (UMAG_04125). UmOps1 and UmOps2 are expressed during the whole life cycle, in axenic cultures as well as *in planta*, whereas UmOps3 was recently shown to be absent in axenic cultures but highly expressed during plant infection. Here we show that expression of UmOps1 and UmOps2 is induced by blue light under control of white collar 1 (Wco1). UmOps1 is mainly localized in the plasma membrane, both when expressed in HEK cells and *U. maydis* sporidia. In contrast, UmOps2 was mostly found intracellularly in the membranes of vacuoles. Patch-clamp studies demonstrated that both rhodopsins are green light-driven outward rectifying proton pumps. UmOps1 revealed an extraordinary pH dependency with increased activity in more acidic environment. Also, UmOps1 showed a pronounced, concentration-dependent enhancement of pump current caused by weak organic acids (WOAs), especially by acetic acid and indole-3-acetic acid (IAA). In contrast, UmOps2 showed the typical behavior of light-driven, outwardly directed proton pumps, whereas UmOps3 did not exhibit any electrogenity. With this work, insights were gained into the localization and molecular function of two *U. maydis* rhodopsins, paving the way for further studies on the biological role of these rhodopsins in the life cycle of *U. maydis*.

## Introduction

Light is a prominent environmental parameter, impacting the life cycle of fungi by regulating the expression of many genes. In the kingdom of fungi, distinct photoreceptors optimized for different light colors have evolved, each consisting of a protein moiety and a light absorbing chromophore. While blue light receptors [cryptochrome-photolyase family, white collar 1 (Wco1), and vivid proteins] use flavin as chromophore, red and far-red light is perceived by biliverdin bound to phytochromes. Green light is absorbed by rhodopsins, opsin proteins carrying a retinal molecule as chromophore (Brown, 2004; Dasgupta et al., 2016).

Fungal rhodopsins are assigned to the large family of microbial rhodopsins, also referred to as type 1 rhodopsins sharing an overall structure similar to animal type 2 rhodopsins despite lack of sequence homology. The basic structure of rhodopsins is a seven transmembrane helix motif with retinal as chromophore covalently linked to a conserved lysine residue via Schiff base (Ernst et al., 2014). In the dark (ground state), the retinal’s conformation is all-*trans*. Upon illumination, the chromophore photoisomerizes to 13-*cis* retinal followed by conformational changes in the whole protein, triggering the appropriate function (Ernst et al., 2014). The first microbial rhodopsin, bacteriorhodopsin (BR), was discovered in the purple membrane of *Halobacterium salinarum* (Oesterhelt and Stoeckenius, 1971). This well-investigated microbial rhodopsin is used as reference for all later discovered rhodopsins that provide diverse distinct features and absorb light of different wavelengths. Microbial rhodopsins include the inwardly directed chloride pump halorhodopsin (HR), two sensory rhodopsins (SRI and SRII), which are involved in the regulation of flagellar movement (Matsuno-Yagi and Mukohata, 1980; Bogomolni and Spudich, 1982; Schobert and Lanyi, 1982; Spudich et al., 1986); inward-rectifying cation channels from the green alga *Chlamydomonas reinhardtii* (Nagel et al., 2002, 2003); anion channels from *Guillardia theta* (Govorunova et al., 2016); an outwardly directed sodium pump (KR2 from *Krokinobacter eikastus*) (Inoue et al., 2013); and an inward proton pump (PoXeR) from the marine bacterium *Parvularcula oceani* (Shevchenko et al., 2017). Due to this high variability in pumping different ions, microbial rhodopsins have gained great importance as optogenetic tools used for neuron activation and silencing, and are used in technical applications as bio-nanomaterial for bio-electronics (Adamantidis et al., 2015; Mahyad et al., 2015; Govorunova and Koppel, 2016).

Up to now, very little is known about rhodopsins from fungi. The distribution of rhodopsins in the fungal kingdom is sporadic and, in addition, various fungi also contain opsin-related proteins (ORPs) besides the green light sensing rhodopsins (Wang et al., 2018). The first fungal rhodopsins discovered were from Ascomycetes, including Nop-1 from *Neurospora crassa* and LR from *Leptosphaeria maculans*. Nop-1 exhibits a slow photocycle similar to photosensors (Bieszke et al., 1999), whereas LR acts as an outward proton pump showing a fast photocycle similar to BR (Waschuk et al., 2005). Based on these properties, a three-type classification of fungal rhodopsins was introduced (Brown and Jung, 2006): NR-like rhodopsins (NR from *N. crassa* rhodopsin) with photosensory characteristics and LR-like rhodopsins (LR from *L. maculans* rhodopsin) exhibiting a fast photocycle with capability of ion pumping. LR-like and NR-like rhodopsins are very similar in their amino acid sequence thus merging in one phylogenetic clade which was recently designated as Nop-1 like rhodopsins (Wang et al., 2018). The third subgroup is represented by the auxiliary ORP-like rhodopsins, later called CarO-like rhodopsins (Brown and Jung, 2006; Wang et al., 2018). ORPs show similar overall structure to BR concerning the transmembrane regions, but do not exhibit the lysine residue for retinal binding via Schiff base. In contrast, CarO-like rhodopsins still exhibit sequence similarity to ORPs but, in addition, contain the important lysine residue, thus representing real rhodopsins. The first CarO-like rhodopsin investigated in detail was PhaeoRD2 from *Phaeosphaeria nodorum* showing a fast photocycle (Fan et al., 2011). CarO of the rice pathogen *Fusarium fujikuroi* was recently shown to act as a green light-driven outward rectifying proton pump (García-Martínez et al., 2015).

Although further putative fungal rhodopsins were discovered in sequenced genomes, just few of them – and only from ascomycetes – have been analyzed biophysically to reveal their physiological function (Brown et al., 2001; Waschuk et al., 2005; Fan et al., 2011; García-Martínez et al., 2015). Moreover, the biological role of rhodopsins in fungi remains mainly unknown. CarO was recently shown to retard the germination of conidia in light (García-Martínez et al., 2015; Brunk et al., 2018). Moreover, CarO showed enhanced pump activity when exposed to weak organic acids (WOAs) like gluconate, glutamate (García-Martínez et al., 2015), acetate, and indole-3-acetic acid (IAA) (Adam et al., 2018). Since IAA is an important plant hormone involved in different responses including growth, its effect on CarO could indicate that CarO-like rhodopsins are associated with plant–fungus interactions. This assumption is supported by the recently discovered involvement of an LR-like rhodopsin, Sop1 from *Sclerotinia sclerotiorum*, in its virulence (Lyu et al., 2015). Wang et al. (2018) suggest that NOP-1 could play a role in regulating the asexual-sexual switch in response to different environmental signals including light and reactive oxygen species (ROS) level in *N. crassa*.

The situation in basidiomycetes is even less clear. There are only a few examples of light responses known so far in the maize pathogen *Ustilago maydis* as a representative of the basidiomycetes. Transcript levels of Um*ops1*, Um*ops2*, as well as of *car1*, the presumed phytoene desaturase, are induced by white light in axenic cultures, and that fungus synthesizes β-carotene (Estrada et al., 2009). The formation of basidiocarps, a rare event in *U. maydis*, is induced by low irradiances of white light (Cabrera-Ponce et al., 2012). It was also shown that blue light induces the expression of photolyase genes in *U. maydis*, and that this response is under control of the Wco1 ortholog, which controls also several other blue light-controlled genes as identified in microarray studies (Brych et al., 2016).

*Ustilago maydis* contains three putative photoactive rhodopsins, called ops1 (UMAG_02629), ops2 (UMAG_00371), and ops3 (UMAG_04125) (Estrada et al., 2009) termed here UmOps1, UmOps2, and UmOps3, respectively. According to the classification of fungal rhodopsins introduced by Brown and Jung (2006), UmOps1 and UmOps3 are similar to CarO-like rhodopsins, while UmOps2 is related to the LR-like rhodopsins. Estrada et al. (2009) showed that UmOps1 and UmOps2 were expressed in axenic cultures whereas they could not detect UmOps3 mRNA under these conditions. This finding was supported by a recent RNAseq-analysis that revealed no UmOps3 expression in axenic cultures (Lanver et al., 2018). In contrast, UmOps3 is expressed during plant infection (Ghosh, 2014) with highest transcript levels observed 12 days after infection of the corn plants (Lanver et al., 2018). Since UmOps3 seems to be only expressed in the biotrophic phase, we focused here on the analysis of UmOps1 and UmOps2.

First, the regulation of expression of UmOps1 and UmOps2 under defined monochromatic light regimes and the hereby involvement of Wco1 and phytochrome 1 (Phy1) were studied. Second, the localization of the rhodopsins in a heterologous expression system as well as in fungal sporidia was investigated with different fluorescence microscopic methods including super-resolution microscopy. In addition, by using the patch-clamp technique, UmOps1 and UmOps2 were functionally analyzed to figure out whether these two rhodopsins respond to light. Action spectra were recorded to reveal the optimal wavelength for activation of these rhodopsins. Further experiments were performed to investigate whether UmOps1 and UmOps2 are outward rectifying proton pumps and whether their pumping activity is influenced by extracellular WOAs.

## Materials and Methods

### Construction of *U. maydis* Mutants

All *U. maydis* strains generated in this study are derived from the wild type isolate FB1 (Banuett and Herskowitz, 1989). For the deletion mutants Δ*wco1* (UMAG_03180), Δ*phy1* (UMAG_05732), Δ*ops1* (UMAG_02629), and Δ*ops2* (UMAG_00371), the open reading frame of the respective genes was substituted by a resistance cassette through homologous recombination. Therefore, about 1 kb of each flanking region was amplified by PCR. Primers are listed in Supplementary Table S1. PCR products were digested with *Sfi*I and ligated to the hygromycin cassette of pMF1-h (Brachmann et al., 2004).

For construction of *U. maydis* strains constitutively expressing an UmOps2-eGFP fusion protein, the gene was cloned into a plasmid (pETEF-GFP-MXN; p1742) containing the *etef*-promoter, the *egfp*-sequence, and a carboxin resistance cassette (Böhmer et al., 2008). Because UmOps1-eGFP expression could not be detected in any of the transformants suggesting a negative effect of UmOps1 on *U. maydis* cells when constitutively expressed, the open reading frame of Um*ops1* was cloned between the arabinose-inducible *crg*-promoter and the *egfp*-sequence of plasmid (pCRG-GFP-MXN; p1747) otherwise identical to p1742 (Böhmer et al., 2008). The same strategy was also used to establish plasmids with Um*ops2*-under control of the crg-promoter. Plasmids were linearized with *Ssp*I and integrated into the *ip* locus (succinate dehydrogenase) of *U. maydis* FB1 (mating type a1 b1) (Banuett and Herskowitz, 1989) wild type or the corresponding deletion mutants.

Transformation of *U. maydis* was performed as described (Tsukuda et al., 1988; Schulz et al., 1990). For selection of transformants, potato dextrose agar (PDA) plates containing 200 μg mL^-1^ hygromycin or 5 μg/mL carboxin were used. All mutant strains were confirmed by PCR and Southern analysis. A list of the strains used throughout this study is provided as Supplementary Table S2.

Sporidia were grown on solid (PDA; 39 g/L) or liquid [100 rpm; potato dextrose broth (PDB); 24 g/L] media at 28°C. Sporidia were stored in 0.5 × PDB supplemented with 4 g/L peptone, 0.5 g/L yeast extract, 2.5 g/L sucrose, and 35% glycerol at -80°C.

### *U. maydis* Opsin Constructs for Mammalian Cell Expression

Full-length Um*ops1* (UMAG_02629) and Um*ops2* (UMAG_00371) were PCR-amplified from *U. maydis* cDNA (Brych et al., 2016) with primers listed in Supplementary Table S1. PCR products were first subcloned in pGM-T (Promega) and thereafter cloned into the vector pcDNA^TM^5/FRT/TO-carO::eYFP (García-Martínez et al., 2015) with *Hind*III (5′-end) and *Not*I (3′-end) in frame with *eyfp* while omitting the *carO* gene. Um*ops3* (UMAG_04125) was synthesized (Genscript) with codon usage optimized for human and cloned with *BamH*I into a modified pcDNA^TM^5/FRT/TO-carO::YFP plasmid exhibiting two *BamH*I restriction sites flanking the *carO* gene, yielding pcDNA^TM^5/FRT/TO-Um*ops3*::eYFP. Um*ops1*D225E and Um*ops1*L149W mutants were produced by site-directed mutagenesis in a PCR reaction using primers given in Supplementary Table S1. After *Dpn*I digestion of the template DNA, the PCR product was transformed into *Escherichia coli* XL1-Blue cells. The plasmids were propagated and purified in small or medium scale (Marcherey Nagel NucleoSpin^®^Plasmid or NucleoBond^®^Xtra Midi EF). All plasmids were sequenced to verify their correctness before their application in further experiments.

### Light Treatment of *U. maydis*

Cells of the FB1 wild type or the deletion mutants Δ*wco1* or Δ*phy1* were grown in YEPS-L medium (0.4% yeast extract, 0.4% peptone, and 2% sucrose) (Tsukuda et al., 1988) at 28°C and dark-adapted by incubation overnight in complete darkness. The following day, cultures were diluted in YNB-SO_4_ medium supplemented with 2% glucose (Mahlert et al., 2006; Freitag et al., 2011) under dim green light. Cultures were split in two aliquots, one as dark control and one for the blue light (471 nm), red light (655 nm), or far-red light (740 nm) treatment each of them with a fluence rate of 10 μmol/m^2^/s applied by LED light sources (CLF Plant Climatics). After 60 min of light treatment or incubation in darkness, cells were harvested for RNA extraction as described (Brych et al., 2016).

### RNA Isolation and qRT-PCR

RNA was extracted as described before (Brych et al., 2016) using a Turbo DNA-Free^TM^ Kit (Invitrogen^TM^ by Thermo Fisher Scientific, Carlsbad, CA, United States); 2 μg of RNA were transcribed with the FastGene^®^Scriptase Basic (Nippon Genetics). Quantitative RT-PCR was performed on a Rotor-GeneQ cycler (Qiagen) using the 2x qPCRBIO SyGreen Mix Separate-ROX Kit (Nippon Genetics). Cycling conditions were 2 min 95°C, followed by 40 cycles of 5 s 95°C, 30 s 60°C, and an increase in temperature from 72 to 95°C for melting analysis. The gene *cpr1* coding for cyclophilin (peptidylprolyl isomerase; UMAG_03726) was used as internal standard. Primer efficiency was tested to be in the range of 95–105%. Primers are listed in Supplementary Table S1.

### Mammalian Cell Culture

Human embryonic kidney (HEK) Flp-In^TM^ T-REx^TM^-293 cells (Thermo Fisher Scientific) were stably transfected with pcDNA^TM^5/FRT/TO-Umops1::eyfp or pcDNA^TM^5/FRT/TO-Umops2::eyfp, to obtain the respective HEK293 cell lines following the manufacturer’s instructions. Stable cell lines were cultured at 37°C and 5% CO_2_ in Dulbecco’s Modified Eagle Medium (DMEM high glucose, 4500 mg/L glucose) that was supplemented with 10% fetal calf serum, 100 U/mL penicillin, 100 μg/mL streptomycin, 15 μg/mL blasticidin, and 100 μg/mL hygromycin B. Cells were grown to a confluency of 70–90%, detached with trypsin-ethylenediaminetetraacetic acid (EDTA) solution (0.05 g/L trypsin, 0.02 g/L EDTA in Hank’s Balanced Salt Solution), and subcultivated in a ratio of 1:10 to 1:20 twice a week. Expression of UmOps1::eYFP or UmOps2::eYFP was initiated by adding 3 μg/mL tetracycline 15–24 h before the start of further experiments. Media were supplemented with 1 μM all-*trans-*retinal to ensure availability of the rhodopsin’s chromophore.

For single turnover experiments and the analysis of UmOps1L149W and UmOps1D225E mutants, NG108-15 cells were transiently transfected as described recently (Feldbauer et al., 2016).

### Patch-Clamp Experiments

Patch-clamp experiments were performed as described in detail before (García-Martínez et al., 2015; Adam et al., 2018). The setup was slightly modified now offering a piezo-driven micromanipulator (SMX Micromanipulator, Sensapex) for adjusting the pipette position. A 532 nm DPSS laser with intensities of at least 10 mW/mm^2^ was used. The composition of the standard patch-clamp solutions was described recently (Adam et al., 2018). For the identification of the transported ion species in the extracellular solution, NaCl was replaced by equimolar concentration of sodium gluconate (absence of chloride). Similarly, in the pipette solution, NaCl was replaced by an equimolar concentration of either CsCl or tetraethylammonium chloride (TEACl). For the analysis of the effects of WOAs, NaCl pH 5.0 or NaGlu pH 5.0 bath solution was supplemented with NaAc pH 5.0, IAA (250 mM stock in ethanol absolute), indole-3-propionic acid (IPA) (250 mM stock in ethanol absolute), or indole only (250 mM stock in ethanol absolute) in various concentrations. A voltage-step protocol was used for recording the *I*–*V* plots. For the analysis of closing kinetics, light pulses of 1 ms duration were applied at 0 mV. The relaxation of pump enhancement after solution exchange was measured at 0 mV with 200 ms illumination every 10 s.

Data were analyzed with ClampFit 10.7 software, Excel, and Origin Pro 2016 64Bit. Mean and standard deviation of the values obtained from different cells were calculated. Current values were normalized to the value obtained at 0 mV clamp voltage in bath solution NaCl pH 7.4 and plotted against the applied membrane voltage.

### Sample Preparation for Fluorescence Microscopy

Staining procedures and imaging of HEK293 cells and sporidia were performed in poly-D-lysine covered 8-well tissue culture chambers [for confocal laser scanning microscopy (CLSM) imaging: eight-well tissue culture chamber, Sarstedt, or eight-well Lab-Tek^®^II Chambered # 1.5 German Coverglass System, Nunc^TM^, Thermo Fisher Scientific; for SIM Imaging: μ-Slide eight-well, ibidi].

HEK293 cells were seeded in a density of about 10^4^ cells per well and grown for 12–24 h. Organelles were stained with below specified staining solutions for 30 min at 37°C and 5% CO_2_. If not stated otherwise, 250 μL staining solution were used per well. Nuclei or nucleic acids, respectively, were visualized by NucRed^TM^ Live 647 ReadyProbes^TM^ Reagent (NucRED; 1–2 drops/mL in growth medium) or 2.5 μM SYTO^®^59 Red Fluorescent Nucleic Acid Stain (Thermo Fisher Scientific) in NaCl patch-clamp solution (NPCS) pH 7.4. Acidic regions were identified with 0.5 μL pHrodo^®^Red AM (Thermo Fisher Scientific) intracellular pH indicator and 5 μL Powerload^TM^ (provided with the AM ester) per mL NPCS pH 7.4. After removal of staining solution, each well was washed three times with 500 μL NPCS pH 7.4 and filled with 300 μL NPCS pH 7.4 for imaging.

*Ustilago maydis* sporidia with the inducible UmOps1::GFP were grown in PDB for 15–24 h at 28°C and 100 rpm, harvested by centrifugation (4000 *g*, 3 min), washed once in ddH_2_O, and resuspended in induction medium (1.7 g/L yeast nitrogen base; 0.2% ammonium sulfate; 2.0% arabinose). After about 3 h, the sporidia were harvested again, washed, and resuspended in ddH_2_O to a final density of 6.5 × 10^5^ sporidia/mL. Sporidia expressing UmOps2::eGFP were likewise grown in PDB and washed, but directly resuspended in water. The sporidia were then seeded in PDL-coated eight-well tissue culture chambers (250 μL/well) and incubated for 30 min at 28°C in presence of white light with intensity of about 5 W/m^2^. After removal of the medium, sporidia were stained with dyes dissolved in ddH_2_O. Nucleic acids were stained with 1.25–2.5 μM SYTO^®^59, a dye which according to the manufactures manual preferentially labels mitochondria in yeast cells. For acidic region labeling, cells were stained with 0.5 μL pHrodo^®^Red AM and 5 μL Powerload^TM^ (Thermo Fisher Scientific) per mL ddH_2_O. Vacuolar membranes were stained with 8 μM FM4-64 Dye (*N*-(3-triethylammoniumpropyl)-4-(6-(4-(diethylamino) phenyl) hexatrienyl) pyridinium dibromide; Thermo Fisher Scientific). Staining was performed at 28°C in the dark for either 30 min (SYTO^®^59, pHrodo^®^Red) or 15 min (FM4-64). Then, sporidia were washed two times with ddH_2_O and imaged in 400 mOsm sorbitol.

### Imaging Procedures

#### Confocal Laser Scanning Microscopy (CLSM)

A confocal laser scanning microscope (SP700, Zeiss, Germany) equipped with three laser lines (488 nm: 10 mW, 555 nm: 10 mW, 639 nm: 5 mW) was used. Images were recorded using a plan-apochromat 63x/1.40 oil M27 objective. Frame size for imaging was set to 1024 × 1024 pixels except otherwise specified, with a bit depth of 16 bit. PMT detector gain for all channels was 500–700, laser powers between 1 and 2.8%, and pixel dwell times from 1.27 to 3.15 μs were used. Every line was averaged from two recordings with the laser scanning unidirectionally. The pinhole was adjusted to 1 airy unit (AU). Images were processed with ZEN software (ZEN 2012, Zeiss) or Fiji, Version ImageJ 1.50f (Schindelin et al., 2012).

#### Structured Illumination Microscopy (SIM)

Structured illumination microscopy (SIM) allows imaging with a resolution below the refraction limit by using a grid illumination pattern (Gustafsson et al., 2008). SIM images were generated using a Zeiss Elyra S.1 SIM (Carl Zeiss AG) equipped with a 63x oil immersion objective (Plan-Apochromat 63x/1.4 Oil DIC M27; Strehl ratio > 90%) and a sCMOS PCO Edge 5.5 camera for recording. Different laser wavelengths were used for the excitation of eGFP (488 nm), FM4-64 (488 or 555 nm), pHrodo^®^Red (555 nm), and SYTO^®^59 (640 nm). Images were processed as for CLSM.

### Protein Alignments and Modeling

Amino acid sequences from BR (gb| AAA72504), CarO (gb| CAD97459), Nop-1 (xp000959421), LR (gb| AAG01180), UmOps1 (UMAG_02629), UmOps2 (UMAG_00371), and UmOps3 (UMAG_04125) were aligned using PSI/TM-Coffee alignment (Di Tommaso et al., 2011). UmOps1 was modeled with Swiss model (Biasini et al., 2014) based on the crystal structure of *Acetabularia* rhodopsin I (5awz.1) (Furuse et al., 2015).

## Results

### Expression of UmOps1 and UmOps2 Is Induced by Light

Expression of Um*ops1* and Um*ops*2 genes is induced by white light (Estrada et al., 2009). However, it was unknown which light qualities contribute to this effect and which photoreceptors are involved. To answer these questions, we treated dark-grown axenically grown FB1 cells of wild type, Δ*wco1*, and Δ*phy1* mutants with monochromatic blue, red, and far-red light of the same fluence rates for 1 h, isolated the RNAs and quantified Um*ops1* and Um*ops2* transcript levels by qRT-PCR.

**FIGURE 1:**
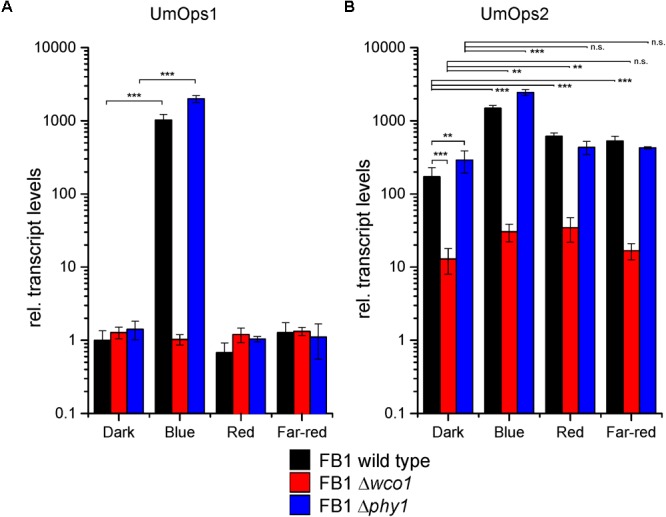
Light regulation of **(A)** Um*ops1* and **(B)** Um*ops2* expression. Transcript levels were quantified by qRT-PCR. FB1 cells were grown in complete darkness and then illuminated with monochromatic lights at a fluence rate of 10 μmol/m^2^/s^1^ for 1 h. Transcript levels were normalized against the one of Um*ops1* in dark-grown wild type and cyclophilin. Shown are means and SD of three biological replicates kept in darkness or illuminated with either blue (λ_max_ 471 nm), red (λ_max_ 664 nm), or far-red (λ_max_ 740 nm) light as indicated. Significance levels between group samples are indicated for *p*-values over 0.05 with not significant (n.s.), for <0.05 with one asterisk (^∗^), for <0.01 with two asterisks (^∗∗^), and for <0.001 with three asterisks (^∗∗∗^). Note that relative transcript levels are given in logarithmic scale.

Um*ops1* transcript levels were essentially the same in all three genotypes in dark-grown cells but about 1000-fold induced after blue light treatment in the wild type and the Δ*phy1* mutant. This induction was completely abolished in the Δ*wco1* mutant. Red and far-red light had no effect in all strains (Figure 1A). Thus, we conclude that Um*ops1* expression is exclusively controlled by Wco1.

Um*ops2* transcript levels were higher than the ones of Um*ops1* already in darkness while the induction rates caused by light treatment were lower compared with Um*ops1*. Surprisingly, the Δ*wco1* mutant showed 13-fold lower Um*ops2* transcript levels than wild type already in dark-grown cells. This finding indicates a role of Wco1 in Um*ops2* expression independent from light (Figure 1B). Likewise, though to much lower extend, also Phy1 seems to have a function independent from light but opposite to Wco1 since the Um*ops2* level was 1.8-fold higher in Δ*phy1* compared to wild type in dark-grown cells.

Despite the role of Wco1 and Phy1 on Um*ops2* expression in darkness, blue, red, and far-red light caused inductions in the wild type of 8.7-, 3.6-, and 3.1-fold, respectively. The blue light induction in wild type was strongly but not completely abolished in the Δ*wco1* mutant (remaining 2.3-fold induction) suggesting involvement of another photoreceptor besides Wco1 in blue light control of Um*ops2*.

Surprisingly, no significant difference was observed between wild type and Δ*phy1* after red and far-red treatments. However, one should consider that the Δ*phy1* mutant exhibited increased transcript levels in the dark that are unaltered after red/far-red illumination, whereas the wild type exhibits a significant increase in Um*ops2* transcripts after red or far-red light-illumination. In contrast, under the same conditions, the Δ*wco1* mutant showed 9.6- and 31.5-fold reduced Um*ops2* levels compared to the wild type under red light and far-red light, respectively, but 2.6-fold and 1.3 fold increased Um*ops2* levels compared to the dark. Together these data suggest that the regulation pattern of Um*ops2* is much more complex than that of Um*ops1*, and that Wco1 plays a role not only in blue light but also in red and far-red light signaling.

### UmOps1 and UmOps2 Are Green Light-Driven Proton Pumps

**FIGURE 2:**
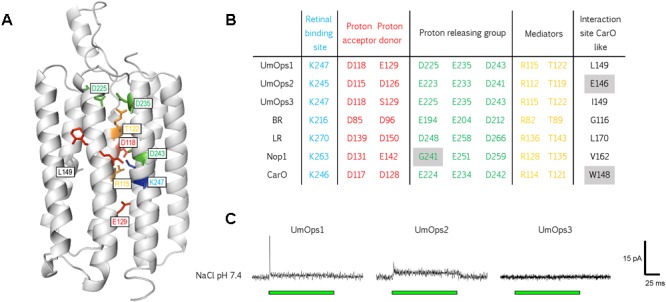
Structural conservation and physiological function of the three *U. maydis* rhodopsins. **(A)** Model of UmOps1 based on the crystal structure of the *Acetabularia* rhodopsin I with resolution of 1.57 Å (5awz.1) (Furuse et al., 2015). **(B)** Prominent residues that are well conserved in microbial rhodopsins of BR, LR, Nop1, CarO, and the three *U. maydis* rhodopsins. The respective position of the amino acids in the rhodopsin entity is given exemplary for UmOps1 in A. The well-conserved proton donor corresponding to Asp-96 in bacteriorhodopsin is replaced by a glutamate in UmOps1 and a serine in UmOps3. **(C)** Whole-cell patch-clamp analysis of the rhodopsins that were heterologously expressed in HEK293 cells. A 532 nm laser (green bar) was used for excitation. While UmOps1 and UmOps2 are green light-activated ion pumps, no light-dependent signal was observed for UmOps3.

To date, there is no study about fungal rhodopsins from basidiomycetes. We performed multiple sequence alignment of the three *U. maydis* rhodopsins, alongside with BR and the three rhodopsins that are eponymous for the respective fungal rhodopsin clade (Brown and Jung, 2006; Wang et al., 2018). We analyzed whether structurally important residues that are required for proton pumping are conserved in the *U. maydis* rhodopsins (Figure 2A,B and Supplementary Figure S1). While UmOps1 and UmOps2 fulfill all requirements for proton pump activity, UmOps3 is most likely a sensory rhodopsin, as the proton donor expected at position 129 (homologous of BR D96) is replaced by a serine, similar as in the *Anabaena* sensory rhodopsin (Jung, 2007). In UmOps2, the proton donor position is filled by a glutamate residue that generally enables the proton transport, especially in proteorhodopsins (Ernst et al., 2014; Olson et al., 2018), but is also present in Nop1, which has a slow photocycle. In contrast, we found the aspartate of the proton acceptor (homologous of BR-D85) to be well conserved in all investigated rhodopsins of *U. maydis*.

The proton releasing group plays an important role in the proton pumping rhodopsins. Nop1 exhibits a glycine (G241) in the same position as BR-E194 hampering the proton release, while in LR, this position is held by an aspartate that still enables the proton release. All three *U. maydis* rhodopsins exhibit intact proton releasing sites with either aspartate (UmOps1) or glutamate (UmOps2 and UmOps3) in positions equivalent to BR-E194. A general feature of all *U. maydis* rhodopsins is the occurrence of leucine in the position of BR-T178 that is in close proximity to a well conserved water molecule in BR and was shown to influence the pumping behavior in BR and HR (Paula et al., 2001). This threonine residue is well conserved in the ascomycete rhodopsins with only a few exceptions (members of Chaetothyriales/Xylariales), where leucine or isoleucine is present at this position (Adam et al., 2018). Though, UmOps1 is most related to the group of CarO-like rhodopsins that is characterized by the occurrence of tryptophan or glutamate at the same position as BR-G116 (Brown and Jung, 2006; Fan et al., 2011), in contrast UmOps1 exhibits a leucine at this position. On the other hand, in UmOps2, the CarO-like characteristic glutamate is present, though the protein is more similar to the LR-like rhodopsins. This finding suggests that the system used for the classification of rhodopsins in ascomycetes might not completely fit the situation in basidiomycetes.

To decipher the physiological function of the *U. maydis* rhodopsins, we expressed UmOps1, UmOps2, and UmOps3 C-terminally tagged with eYFP in mammalian cells and analyzed their response to green light by means of whole-cell patch-clamp techniques. The characterization of the *U. maydis* rhodopsins was performed in HEK293 cells exhibiting a single gene copy located at a certain locus (FRT site) under control of a tetracycline-inducible promoter. For turn-over measurements, NG108-15 cells were transiently transfected providing higher expression levels.

UmOps1 and UmOps2 showed green light-driven pump activity, while UmOps3 did not exhibit any electrogenity (Figure 2C). It cannot be completely excluded but is very unlikely that the heterologous expression in mammalian cells and the presence of a C-terminal eYFP tag might have altered the protein function in comparison to its native environment. Due to the absence of any electrophysiological signal, UmOps3 was not further investigated in this study. Upon illumination, UmOps1 and UmOps2 evoked an outward-directed current, exhibiting the typical behavior of ion-pumping rhodopsins (Figure 2C). After a fast rise to a transient maximum, the photocurrent decreased to a positive stationary value. While in UmOps1 at pH 7.4, the transient is well pronounced, in contrast in UmOps2, the transient is only hardly detectable. After short-pulse illumination (1 ms) in both rhodopsins, the pump current decayed in a biexponential manner exhibiting a fast (UmOps1: τ_off-1_ = 0.91 ± 0.22 ms, mean and SD from nine experiments; UmOps2: τ_off-1_ = 1.5 ± 0.6 ms; five experiments) and a slower time constant (UmOps1: τ_off-2_ = 90 ± 23; UmOps2: τ_off-1_ = 9.2 ± 1.4). The current amplitude obtained from UmOps1 (0.032 ± 0.016 pA/pF, mean and SD from 12 experiments) and UmOps2 (0.069 ± 0.027 pA/pF, mean and SD from nine experiments) at neutral pH was relatively low in comparison to previous experiments with other rhodopsins such as CarO (2.2 ± 0.6 pA/pF) (García-Martínez et al., 2015).

**FIGURE 3:**
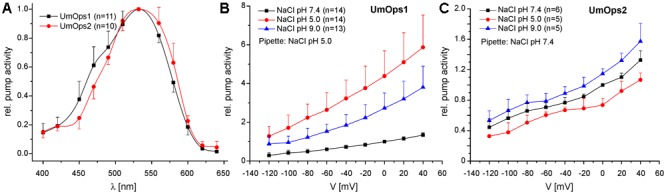
Electrophysiological characterization of UmOps1 and UmOps2. **(A)** Action spectra of UmOps1 and UmOps2. The mean relative pump activity and standard deviation of *n* measurements as indicated are given in dependence of the wavelength used for excitation. Both rhodopsins show highest activation at 532 nm. In these measurements, NaCl pH 5.0 was used as pipette solution to support proton pumping at low light intensities. Current–voltage relation of UmOps1 **(B)** and UmOps2 **(C)** in a range of +40 to –120 mV after excitation with a 532 nm laser in different extracellular solutions as indicated. Shown is relative pump activity (mean + standard deviation of *n* measurements as indicated) normalized to the value obtained in bath solution NaCl pH 7.4 at 0 mV clamp voltage. Note that pump activity of UmOps1 is highest at extracellular pH 5.0, which is remarkable for an outward directed proton pump, as the gradient is working against the pump.

We analyzed the spectral range promoting maximal pump activity by recording the action spectrum. UmOps1 as well as UmOps2 yielded bell shaped action spectra ranging from 400 to 640 nm (Figure 3A) with the maximal pump activity in the green spectral range (excitation with 532 nm). The action spectrum of UmOps2 was slightly red-shifted in comparison to the one of UmOps1.

To figure out, whether UmOps1 and UmOps2 function as proton pumps, we investigated the voltage and pH dependencies of UmOps1 and UmOps2. A voltage step protocol ranging from -120 to +40 mV was applied while varying the pH values on the extracellular side. Both rhodopsins exhibit positive pump currents at all applied clamp voltages and pH values fulfilling the requirement of an ion pump. For an outwardly directed proton pump, according to the electrochemical gradient, pump activity is expected to be supported at pH 9.0 and diminished at pH 5.0. However, as depicted in Figure 3B,C, the pH dependency highly differed between UmOps1 und UmOps2. While for both rhodopsins current signals increased when extracellular pH 9.0 was applied, UmOps1 and UmOps2 differed in their response to extracellular pH 5.0. At this pH, UmOps2 showed the expected behavior, yielding (slightly) decreased current signals compared to those measured in pH 7.4 solution. In contrast, UmOps1 signals measured at pH 5.0 even excelled the ones at pH 9.0.

At first glance, this result might be judged to be contradictory to the assumption that UmOps1 functions as an outward proton pump. Therefore, the ion species that are transported by UmOps1 and UmOps2 were further investigated and the rhodopsins were tested for inward transport of sodium or outward transport of chloride ions. For UmOps1 and UmOps2, similar results were obtained when measuring with intracellular NaCl pH 5.0 or CsCl pH 5.0, respectively (Supplementary Figure S2). Similarly, when sodium gluconate-based extracellular solutions (NaGlu) of different pH values were used, pump currents were obtained exhibiting unaltered voltage dependency (Supplementary Figure S3). UmOps1 showed even slightly increased pump activity in the absence of chloride. At 0 mV, pump activity increased by a factor of 1.4 ± 0.38 (pH 7.4) and 1.7 ± 0.54 (pH 5.0) in sodium gluconate in comparison to NaCl-based solutions. In UmOps2, the supporting effect of gluconate was almost negligible with a factor of 1.25 ± 0.07 (pH 7.4) and 1.08 ± 0.22 (pH 5.0). Similar as observed in NaCl solutions, UmOps1, but not UmOps2, yielded current signals in NaGlu pH 5.0 higher than in NaGlu pH 9.0, which is consistent with the pH-induced support of pump activity in NaCl bath solutions (Figure 3B and Supplementary Figure S3). Overall, these experiments yielded clear evidence for protons as the transported ion species.

### Pump Activity of UmOps1 but Not UmOps2 Is Augmented by the Auxin IAA

**FIGURE 4:**
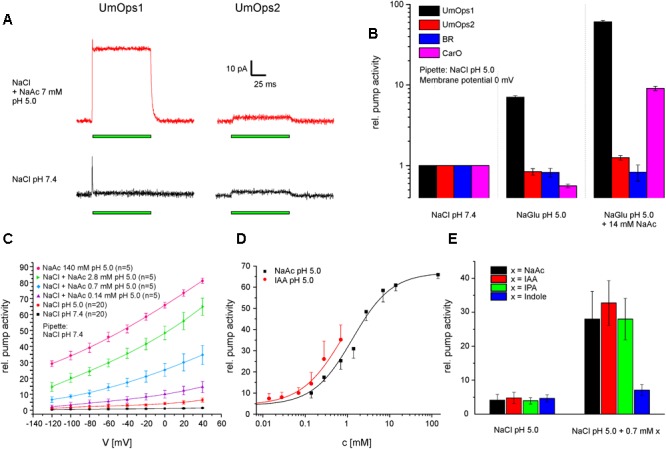
Effect of WOAs on the pump activity of *U. maydis* rhodopsins. **(A)** Typical whole cell traces showing the light-induced charge transfer by UmOps1 and UmOps2 (time of illumination is indicated by the green bar). The pump intensity of UmOps1 strongly increased in the presence of weak organic acids at external pH 5.0 (upper raw) compared to NaCl pH 7.4 (lower raw), while no such effect could be observed with UmOps2. **(B)** Mean relative pump activity and standard deviation of at least five experiments of UmOps1 and UmOps2 in comparison with BR and CarO (data were obtained from Adam et al., 2018) at 0 mV holding potential in NaCl pH 7.4, NaCl pH 5.0, and NaCl pH 5.0 supplemented with 14 mM sodium acetate. Note that only UmOps1 (similar to CarO) shows a clear response to acetate while UmOps2 does not. **(C)** Current–voltage relation of UmOps1 light-induced pump current in the presence of different concentrations of sodium acetate. **(D)** Dose-response of UmOps1 pump activity in sodium acetate and IAA. The sodium acetate data were described by a Hill function, revealing a *k*-value of 1.32 ± 0.18 mM. **(E)** Mean current and standard deviation of at least five experiments of UmOps1 pump activity in NaCl pH 5.0 and after adding 0.7 mM of different WOAs as indicated. Only the compounds with an acetate group showed a strong supporting effect on the pump activity.

In consistence with the fact that UmOps1 shows highest similarity with CarO-like rhodopsins, we noticed that WOAs have also a strong supportive effect (Adam et al., 2018) on the pump activity of UmOps1, whereas it is very weak in UmOps2 (Figure 4A). In these experiments, the gluconate in the bath solution (pH 5.0) was gradually replaced by acetate. At an extracellular concentration of 14 mM acetate, the UmOps1 pump signal increased by a factor of 8.6 (60.9 compared to NaCl pH 7.4) while UmOps2 pump activity only increased by the factor 1.5 (1.25 compared to NaCl pH 7.4) (Figure 4B). Under these conditions, it was shown recently, that BR pump signal was unaltered (0.83 compared to NaCl pH 7.4), while the fungal rhodopsin CarO showed strong enhancement by a factor of 16.2 (9.1 compared to NaCl pH 7.4) (Adam et al., 2018).

At pH 5.0, we expect 44.5% of the acetate molecules to be protonated according to the pKa of 4.76, and acetic acid is highly membrane-permeable. In an unbuffered cellular system, acidic acid can be used to manipulate the pH of the cytosol. On the other hand, in our patch-clamp system, the cytosol is replaced with the pipette solution that is buffered to pH 7.4 with 10 mM HEPES. However, we found the supporting effect to be still present at extracellular pH 7.4, where 99.8% of the acetate molecules are supposed to be deprotonated and thus cannot permeate the membrane. Furthermore, UmOps2 and BR (Adam et al., 2018) do not show altered pump behavior in presence of WOAs (Figure 4B). Thus, we conclude that the supporting effect by WOAs reflects a specific feature of CarO-like rhodopsins, including UmOps1. Strikingly, if the pipette solution at neutral pH is supplemented with 2.8 mM sodium acetate, the supporting effect of WOAs remains similar as in absence of acetate, indicating that the interaction between WOAs and protein does not take place intracellularly (Supplementary Figure S4).

We therefore further investigated the dose-response of UmOps1 to different WOAs (NaAc, IAA, and IPA, pH 5.0). Sodium acetate pH 5.0 was applied to chloride based bath solution in concentrations ranging from 0.14 to 140 mM (Figure 4C). With increasing concentrations of extracellular sodium acetate pH 5.0, the pump activity of UmOps1 increased, showing a dose dependency that was described by a Hill fit, revealing a Hill coefficient of 0.93 ± 0.08, suggesting almost non-cooperative binding, and a maximal relative pump activity at *I*_max_ = 66.9 ± 1.5 (factor of enhancement in comparison to bath solution NaCl pH 7.4) and a half maximal intensity at a concentration of 1.32 ± 0.18 mM (Figure 4D). As the WOAs IAA and IPA are only hardly solvable in aqueous solutions, reliable experimental conditions were limited to a range from 14 μM to 0.7 mM (Supplementary Figure S5). At a concentration of 0.7 mM and 0 mV clamp voltage, a strong increase of pump activity was observed (Figure 4E) with IAA (6.8-fold in comparison to pure NaCl pH 5.0) and IPA (6.8-fold), which is similar to the one observed with sodium acetate (7.1-fold). In contrast, indole alone yielded only a weak effect on the pump activity (1.5-fold), suggesting that the acetate group is the compound that is responsible for the supporting effect of the tested WOAs.

We found the supporting effect of WOAs to be reversible after removal of the WOAs. When UmOps1 was exposed to NaCl pH 9.0 after treatment with sodium acetate pH 5.0, the pump current initially recovered to the initial values with a time constant of 89.2 ± 1.6 s (Supplementary Figure S6). Though the decay of the signal is similar to the one described for CarO (Adam et al., 2018), an initial transient increase of the pump activity in pH 9.0 as described for CarO was absent in UmOps1 (Supplementary Figure S6). In CarO, this transient increase in pump activity after the jump from pH 5.0 to pH 9.0 was hypothesized to be the sum of the effects induced by the increased pH gradient (increase of pump activity) and removal of WOAs interaction with the rhodopsin (decrease of pump activity) (García-Martínez et al., 2015). We presumed a potential role of the proton releasing site in the interaction with the WOAs. In UmOps1, the BR194 proton releasing site is represented by an Asp whereas by a Glu in CarO. Indeed, the UmOps1 D225E mutant showed a transient increase upon pH shift to pH 9.0 similar as in CarO relaxing with a similar time constant as the wild type (89.7 s). We also tested for the influence on WOAs interaction of a further site that is characteristic for CarO-like rhodopsins. In CarO-like rhodopsins, the position represented by CarO W148 was assumed to be a potential interaction site with other proteins (Fan et al., 2011). Interestingly, in UmOps1, the position is represented by a Leu (L149). After replacement with Trp, the UmOps1 L149W showed much faster relaxation in pH 9.0 (56.5 s; Supplementary Figure S6).

### UmOps1 and UmOps2 Localize to Different Membranes

**FIGURE 5:**
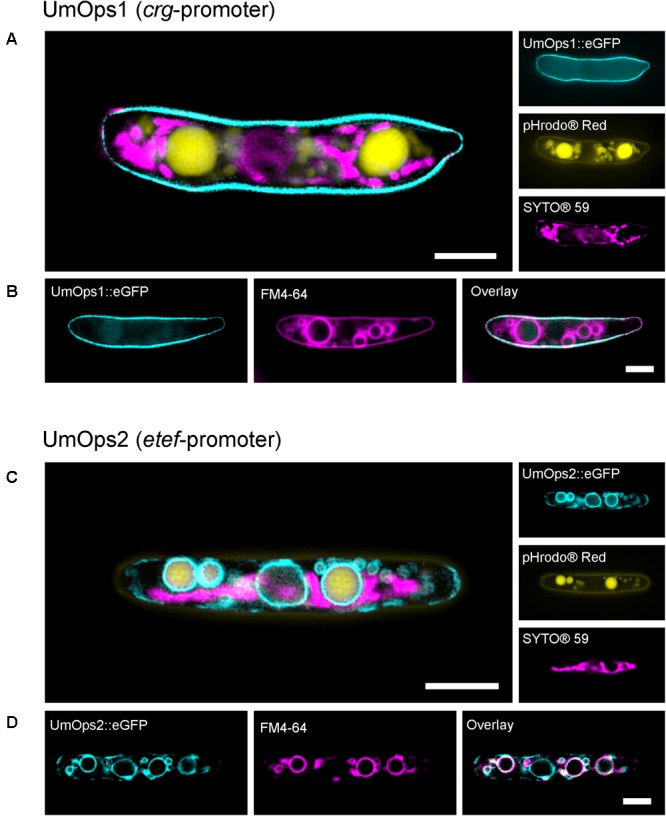
Life-cell fluorescence microscopic analysis of the localization of eGFP-tagged UmOps1 **(A,B)** and UmOps2 **(C,D)** after heterologous expression in *U. maydis* sporidia. Images were obtained with a 3D-SIM. Sporidia were either co-stained for mitochondria (SYTO^®^59) and vacuoles (pHrodo^®^Red; **A,C**), or for vacuolar membranes (FM4-64; **B,D**). Expression of UmOps1-eGFP was driven by the arabinose-inducible *crg*-promoter, expression of UmOps2-eGFP by the constitutive *etef*-promoter. Localization of UmOps2-eGFP after arabinose-induced expression is shown in Supplementary Figure S8. Note that UmOps1 is mainly located in the plasma membrane whereas UmOps2 is present in the vacuolar membranes. Scale bars represent 3 μm.

Knowing that UmOps1 and UmOps2 both represent functional proton pumps, one may assume a potential contribution to the proton motive force alongside the fungus, which would require the presence of the rhodopsins in the plasma membrane. To check the localization of the rhodopsins, *U. maydis* sporidia expressing the rhodopsins as eGFP fusion proteins were investigated with CLSM and SIM (Figure 5). As UmOps3 is only expressed in the biotrophic phase (Ghosh, 2014; Lanver et al., 2018), this protein was excluded from the localization studies.

We found UmOps1 to be located in the plasma membrane and absent in internal membranes (Figure 5A,B). In contrast, UmOps2 is absent in the plasma membrane and mainly localized in internal membranes (Figure 5C,D and Supplementary Figure S8). The eGFP signal of UmOps2 co-localizes with the dye FM4-64, a specific marker for vacuolar membranes (Figure 5D) (Shoji et al., 2006). Depending on the expression level, the rhodopsins are also observed in the endoplasmic reticulum, which is plausible as the ER is involved in the plasma membrane targeting. Nevertheless, UmOps2 was never observed in the plasma membrane independently of the expression level (Supplementary Figure S8; see also Supplementary Table S2). These results are in accordance with our CLSM analysis of HEK293 cells, heterologously expressing the *U. maydis* rhodopsins tagged with eYFP (Supplementary Figure S7). The low amount of UmOps2 in the plasma membrane is in accordance with the low pump currents observed for this rhodopsin (Figure 2C).

## Discussion

The biological role of fungal rhodopsins is not yet well understood, though green light perception is common in fungal species (Fischer et al., 2016; Fuller et al., 2016). First insights into the physiological and regulatory functions of these green light-sensing proteins were obtained recently (García-Martínez et al., 2015; Lyu et al., 2015; Adam et al., 2018; Wang et al., 2018), suggesting a role in the plant–fungus interaction. Herein we used a well-established model organism for biotrophic fungus–plant interaction, the basidiomycete *U. maydis*, which infects corn plants and leads to the formation of tumors on all aboveground parts of the plant (Djamei and Kahmann, 2012). We analyzed the regulation by light of two *U. maydis* rhodopsins, UmOps1 and UmOps2, their electrophysiological function and their localization in sporidia to gain insights into their biological function. To our best of knowledge, this also is the first study on rhodopsins in basidiomycetes.

In *U. maydis*, the expression of Um*ops1* and Um*ops2* was previously found to occur in axenic cultures and to be induced by white (Estrada et al., 2009) and blue light (Brych et al., 2016), whereas Um*ops3* expression is restricted to the biotrophic phase (Ghosh, 2014; Lanver et al., 2018).

The light regulation of Um*ops1* and Um*ops2* in axenic cultures was addressed in more detail in the present study including the analysis of Δ*wco1* and Δ*phy1* mutants. It was found that Um*ops1* expression is strongly induced by blue light but unaffected by red and far-red light, suggesting that only a blue light photoreceptor is involved in the regulation of Um*ops1* expression. Indeed, blue light induction of Um*ops1* was completely absent in Δ*wco1* which is consistent with our previous conclusion that Wco1 is the main blue light photoreceptor in *U. maydis* (Brych et al., 2016). The light regulation of Um*ops2* expression is more complex than that of Um*ops1*, because blue light but also red and far-red light treatment caused an increase in the Um*ops2* transcript level (Figure 1). From the similar behavior of wild type and the Δ*phy1* mutant at these wavebands, we conclude that Phy1 has only a minor role in the light regulation of Um*ops2* while Wco1 is required for the full response to these wavebands. Further studies on the interaction of Wco1 and Phy1 in *U. maydis* are needed to see whether both photoreceptors act together as in *Aspergillus nidulans* (Fischer et al., 2016).

Effects of green light on the expression of the rhodopsins were not elucidated because fungal phytochromes can to some extent also be activated by green light due to their spectral properties (Brandt et al., 2008). Thus, a putative green light effect on UmOps expression does not ultimately tell us which photoreceptor is involved. The same holds true for the LOV-domain containing Wco1. Although the spectral properties of *U. maydis* Wco1 have not been elucidated so far, and published absorption/fluorescence emission spectra of other white collar photoreceptors were not analyzed in the region above 500 nm (He et al., 2002), there is substantial evidence that other LOV-domain photoreceptors absorb to some extent also above 500 nm (Losi et al., 2002). On the other hand, blue light (471 nm) also activates fungal rhodopsins (Figure 3A), making it difficult to decipher the role of rhodopsins in expression regulation without the use of appropriate deletion mutants. This will be the major task of future work, which will include the study of green light effects in *U. maydis* wild type and Δ*ops* mutants. Nevertheless, the strong induction of expression in particular of UmOps1 by Wco1 let us assume that no other photoreceptor, in particular no rhodopsin that has an action spectrum peak around 530 nm (Figure 3A), is involved in regulation of opsin expression (autoregulation).

In contrast to Um*ops1*, the expression of Um*ops2* was repressed in Δ*wco1* already in darkness, indicating a function of Wco1 independent of light. A similar light-independent role of the Wco1 homologue BWC1 of *C. neoformans* was found for the virulence of this basidiomycete (Idnurm and Heitman, 2005).

The Um*ops1* and Um*ops2* genes are not only upregulated in response to light, but the presence of the well conserved lysine residue in all *U. maydis* opsins (K247 in UmOps1; K245 in UmOps2, K247 in UmOps3) also suggests light-responsive behavior of these three proteins. As a requirement, *U. maydis* is capable of producing retinal (Estrada et al., 2009). Furthermore, multiple sequence alignment revealed that, except some slight differences, all residues important for proton pump function are present in the rhodopsins UmOps1 and UmOps2 (Figure 2B and Supplementary Figure S1).

Indeed, our electrophysiological analysis using the patch-clamp technique revealed that both rhodopsins are responsive to green light (peak in action spectrum at 532 nm) with typical characteristics of an outward-directed proton pump (Chow et al., 2010; Fan et al., 2011; García-Martínez et al., 2015) at neutral and alkaline extracellular pHs. While UmOps2 showed reduced pump activity at pH 5, the same condition leads to an unexpected behavior of the UmOps1 rhodopsin, now exhibiting higher pump activity than at neutral/alkaline pH despite the increased proton gradient acting against the pumping direction (Figure 3A). It should nevertheless be considered that UmOps1 under physiological conditions (bath and pipette solution NaCl pH 7.4), yielded very low current signals (0.032 ± 0.016 pA/pF).

Though at first glance the behavior of UmOps1 does not fit the expected pH dependency for proton-pumping rhodopsins, our data clearly show, however, that for both *U. maydis* rhodopsins under these experimental conditions protons are the only ion species to be transported (Supplementary Figures S2, S3). In these measurements, intracellular sodium was replaced by cesium ions and extracellular chloride by gluconate without notably influencing the pump activity (Supplementary Figures S2, S3). The behavior of UmOps1 in acidic conditions is remarkable and such a behavior was not yet reported for other microbial rhodopsins. One may speculate that low extracellular pH causes structural rearrangements in the whole protein or especially at the proton releasing site and hence, leads to facilitated pumping.

Also the supporting effect of WOAs was mainly observed in UmOps1, but hardly seen in UmOps2. In UmOps1, the pump activity was supported in a concentration-dependent manner by the presence of acetate, IAA, and IPA (Figure 4), whereas indole alone, which is a chemical moiety of IAA and IPA, did not provoke a similar reaction of UmOps1 (Figure 4E and Supplementary Figure S5).

When sodium acetate is extracellularly applied at pH 5.0, about one-third of the acetate molecules are protonated and thus are capable of diffusing freely through the plasma membrane with a membrane permeability of 6 × 10^-4^/cm/s (Li et al., 2011). In undefined intracellular lumen, the acetic acid might release protons and by that decrease the intracellular pH. Nevertheless, in our measurements, the cytosol was constantly replaced by the pipette solution (Oliva et al., 1988) and buffered to pH 7.4 with 10 mM HEPES, thus trapping protons eventually released by entering acid molecules. In accordance, we found that under similar experimental conditions the supporting effect of WOAs was present in UmOps1 and CarO, but absent in UmOps2 and BR (Figure 4B; Adam et al., 2018). Therefore, we conclude that this WOA effect is due to protein characteristics rather that unspecific intracellular acidification by acetate. This assumption is further supported by the fact that at neutral pH (pH 7.4 in bath and pipette solution), when almost all acetate deprotonated, the supporting effect by WOAs is still present albeit smaller (Supplementary Figure S4).

A reliable explanation for this pump boosting effect would be a possible interaction of WOAs with the proton releasing group. WOAs might interact with the water network in the rhodopsin or lead to conformational changes or both, resulting in proton pump enhancement. The enrolment of the proton releasing is suggestive from the fact, that the UmOps1D225E (analogous to BR-D194) mutant lacks the increase in pump activity in response to a pH shift from 7.4 to 5.0 and exhibits a reduced response to extracellular WOAs. When after exposure to WOAs the solution is exchanged for WOA-free solution at pH 9.0, the wild type UmOps1 signal remains still enhanced for several minutes, relaxing with a time constant of nearly 100 s (Supplementary Figure S6) suggesting a relatively intense binding of the WOAs to the rhodopsins. In contrast, in UmOps1D225E during the pH 9.0-induced relaxation, an additional transient increase of the pump current was observed (Supplementary Figure S6) as described for CarO from *F. fujikuroi* (García-Martínez et al., 2015; Adam et al., 2018), whereas the relaxation time constant was similar as in the WT protein. Among the CarO-like rhodopsins, the position BR-G116 is typically represented by a tryptophan or glutamate, but by a leucine in UmOps1. This site was suggested to be involved in the interaction with a potential transducer (Fan et al., 2011). Replacement of UmOps1-Leu149 by tryptophan did not provoke a clear effect on the pumping behavior, but strongly influenced the WOAs-supporting effect, revealing a much faster pH 9.0-induced relaxation with a time constant of 59 ± 2 s (Supplementary Figure S6). Thus, based on these initial findings, one may speculate that the supporting effect is due to both, the support of proton release and the stabilization of a more active form of the rhodopsin. Further investigations will be required to understand the mechanisms underlying the supporting effect of acetate in CarO-like rhodopsins.

There is some indication for a potential biological function of the supporting effect of sodium acetate and IAA on the rhodopsin activity. A green-light responsive proton pump that is activated by auxins and WOAs might be of importance for plant-associated fungi including *U. maydis* during host infection. Green light is prevalent in the phyllosphere as light in other spectral ranges is absorbed by photosynthetic pigments (Smith et al., 1990). Indeed, recent investigations give indication for a role of rhodopsins in the fungus–plant interaction, to either damp or support the aggressiveness of the fungus. This assumption is based on current data showing that the absence of CarO in *F. fujikuroi* leads to increased disease symptoms in rice plants (Adam et al., 2018). In contrast, Lyu et al. (2015) showed that the Nop-1 like rhodopsin Sop1 from *S. sclerotiorum* is upregulated during infection and involved in the development of severe disease in crops. A very recent RNAseq analysis revealed that Um*ops1* and Um*ops2* are strongly upregulated early (1 dpi) in the biotrophic phase both with about 15-fold induction compared to axenic cultures (Lanver et al., 2018). At the first glance, it appears to be contradictory that *U. maydis* Δ*cco1* mutants which are unable to produce the opsin’s chromophore retinal did not show any virulence phenotype in corn infection experiments (Estrada and Avalos, 2009). Nevertheless, plants produce all-*trans*-retinal (Lorenzi et al., 1994), which might act as a source for the fungus providing functional rhodopsins during infection. In summary, we presume that UmOps1 and UmOps2 may well have a role in the biotrophic phase, and we will investigate this issue in the future.

Indole-3-acetic acid is an essential plant hormone involved among other responses in growth, cell division and tropism (Leyser, 2018). Also, IPA and indole-3-butyric acid (IBA) are present in plants as precursors of IAA. Typical IAA concentrations in plant sap are in the one digit μM range, but local concentrations of IAA up to 50 μM are reported (Petersson et al., 2009). Moreover, *U. maydis* itself is capable of producing IAA in high concentrations of several hundreds of μM, at least in the tumor tissue on the plant (Moulton, 1942; Turian and Hamilton, 1960; Sosa-Morales et al., 1997; Reineke et al., 2008). Also acetate is present in the plant sap with concentrations of up to 422 μM (Gabriel et al., 1999). In patch-clamp experiments, we found half maximal intensity at a concentration of 1.32 ± 0.18 mM (Figure 4D) acetate, while half maximal intensity values for IAA and IPA could not be determined due to technical issues but are expected in the sub-millimolar range. Indeed, UmOps1 showed slight increase in pump activity already at concentrations of 14 μM and therefore it is a realistic scenario that the regulation of UmOps1 by WOAs plays a role in the plant–fungus interaction.

Beside the fact that a light-driven proton pump is of benefit for general aspects of fungal physiology like the maintenance of the proton-motive force, ATP preservation, and uptake of micronutrients from the environment, rhodopsins might also be involved in the pH regulation of the fungus. It is well known that the environmental pH plays an important role during plant infection (Caracuel et al., 2003; Fernandes et al., 2017).

Interestingly, the rhodopsins do not only differ in the physiological function, but also in their distribution pattern in the sporidia. The rhodopsin UmOps1 showing strong pump activity and reacting to alternating concentrations of extracellular WOAs is mainly located in the plasma membrane. In contrast, UmOps2 that does not exhibit a similar behavior is mostly localized intracellularly in the vacuolar membranes but absent in the plasma membrane (Figure 5). To our best knowledge, there is no report on a rhodopsin localized in the vacuolar membrane. However, there is indication for a functional localization of UmOps2 in the vacuolar membrane. First the protein is trafficked to the vacuolar membrane independent of the expression time and intensity (Supplementary Figure S8). Second, the accumulation of the UmOps2-eGFP signal after arabinose-induced gene expression would not be consistent with a localization within the vacuole where the protein is expected to be degraded (Feyder et al., 2015). However, as a 7TM protein, the tonoplast is the most likely site for UmOps2. The functional expression of the rhodopsin would imply the C-terminus to be oriented outside of the vacuole toward the cytosol. Indeed, the strong fluorescence of the C-terminal eGFP-tag supports the idea that the tag is located in the neutral cytosol but not the acidic vacuole, where the eGFP fluorescence is strongly reduced at low pHs (Kneen et al., 1998). In addition, amino acid sequence analysis by BUSCA (Savojardo et al., 2018) predicted UmOps1 to be a plasma membrane protein while UmOps2 was predicted to be located in intracellular membranes.

The puzzling question, if and how the two rhodopsins interact during fungal growth and how they influence the fungal physiology, will be task of future investigations.

## Author Contributions

AlB and UT conceived this study, supervised the experiments, and wrote the manuscript. AnB conducted qRT-PCR experiments. AnB and AlB interpreted the expression profile. SP performed patch-clamp experiments. SP and UT analyzed the electrophysiological data. SP and AnB performed super resolution microscopy. UT designed the figures. All authors provided discussions and contributed to the manuscript.

## Conflict of Interest Statement

The authors declare that the research was conducted in the absence of any commercial or financial relationships that could be construed as a potential conflict of interest.

## References

[B1] Adam A. Deimel S. Pardo-Medina J. García-Martínez J. Konte T. Limón M. C. (2018). Protein activity of the *Fusarium fujikuroi* rhodopsins CarO and OpsA and their relation to fungus-plant interaction. *Int. J. Mol. Sci.* 19:E215. 10.3390/ijms19010215 29324661 PMC5796164

[B2] Adamantidis A. R. Arber S. Bains J. S. Bamberg E. Bonci A. Buzsáki G. (2015). Optogenetics: 10 years after ChR2 in neurons—views from the community. *Nat. Neurosci.* 18 1202–1212. 10.1038/nn.4106 26308981

[B3] Banuett F. Herskowitz I. (1989). Different a alleles of *Ustilago maydis* are necessary for maintenance of filamentous growth but not for meiosis. *Proc. Natl. Acad. Sci. U.S.A.* 86 5878–5882. 10.1073/pnas.86.15.5878 16594058 PMC297734

[B4] Biasini M. Bienert S. Waterhouse A. Arnold K. Studer G. Schmidt T. (2014). SWISS-MODEL: modelling protein tertiary and quaternary structure using evolutionary information. *Nucleic Acids Res.* 42 W252–W258. 10.1093/nar/gku340 24782522 PMC4086089

[B5] Bieszke J. A. Spudich E. N. Scott K. L. Borkovich K. A. Spudich J. L. (1999). A eukaryotic protein, NOP-1, binds retinal to form an archaeal rhodopsin-like photochemically reactive pigment. *Biochemistry* 38 14138–14145. 10.1021/bi9916170 10571987

[B6] Bogomolni R. A. Spudich J. L. (1982). Identification of a third rhodopsin-like pigment in phototactic Halobacterium halobium. *Proc. Natl. Acad. Sci. U.S.A.* 79 6250–6254. 10.1073/pnas.79.20.6250 6959114 PMC347098

[B7] Böhmer C. Böhmer M. Bölker M. Sandrock B. (2008). Cdc42 and the Ste20-like kinase Don3 act independently in triggering cytokinesis in *Ustilago maydis*. *J. Cell Sci.* 121 143–148. 10.1242/jcs.014449 18089648

[B8] Brachmann A. König J. Julius C. Feldbrügge M. (2004). A reverse genetic approach for generating gene replacement mutants in *Ustilago maydis*. *Mol. Genet. Genomics* 272 216–226. 10.1007/s00438-004-1047-z 15316769

[B9] Brandt S. von Stetten D. Günther M. Hildebrandt P. Frankenberg-Dinkel N. (2008). The fungal phytochrome FphA from *Aspergillus nidulans*. *J. Biol. Chem.* 283 34605–34614. 10.1074/jbc.M805506200 18931394 PMC3259886

[B10] Brown L. S. (2004). Fungal rhodopsins and opsin-related proteins: eukaryotic homologues of bacteriorhodopsin with unknown functions. *Photochem. Photobiol. Sci.* 3 555–565. 10.1039/b315527g 15170485

[B11] Brown L. S. Dioumaev A. K. Lanyi J. K. Spudich E. N. Spudich J. L. (2001). Photochemical reaction cycle and proton transfers in *Neurospora rhodopsin*. *J. Biol. Chem.* 276 32495–32505. 10.1074/jbc.M102652200 11435422

[B12] Brown L. S. Jung K. H. (2006). Bacteriorhodopsin-like proteins of eubacteria and fungi: the extent of conservation of the haloarchaeal proton-pumping mechanism. *Photochem. Photobiol. Sci.* 5 538–546. 10.1039/b514537f 16761082

[B13] Brunk M. Sputh S. Doose S. Van De Linde S. Terpitz U. (2018). HyphaTracker: an ImageJ toolbox for time-resolved analysis of spore germination in filamentous fungi. *Sci. Rep.* 8:605. 10.1038/s41598-017-19103-1 29330515 PMC5766585

[B14] Brych A. Mascarenhas J. Jaeger E. Charkiewicz E. Pokorny R. Bölker M. (2016). White collar 1-induced photolyase expression contributes to UV-tolerance of *Ustilago maydis*. *Microbiologyopen* 5 224–243. 10.1002/mbo3.322 26687452 PMC4831468

[B15] Cabrera-Ponce J. L. León-Ramírez C. G. Verver-Vargas A. Palma-Tirado L. Ruiz-Herrera J. (2012). Metamorphosis of the basidiomycota *Ustilago maydis*: transformation of yeast-like cells into basidiocarps. *Fungal Genet. Biol.* 49 765–771. 10.1016/J.FGB.2012.07.005 22921263

[B16] Caracuel Z. Roncero M. I. G. Espeso E. A. González-Verdejo C. I. García-Maceira F. I. Di Pietro A. (2003). The pH signalling transcription factor PacC controls virulence in the plant pathogen *Fusarium oxysporum*. *Mol. Microbiol.* 48 765–779. 10.1046/j.1365-2958.2003.03465.x 12694620

[B17] Chow B. Y. Han X. Dobry A. S. Qian X. F. Chuong A. S. Li M. J. (2010). High-performance genetically targetable optical neural silencing by light-driven proton pumps. *Nature* 463 98–102. 10.1038/Nature08652 20054397 PMC2939492

[B18] Dasgupta A. Fuller K. K. Dunlap J. C. Loros J. J. (2016). Seeing the world differently: variability in the photosensory mechanisms of two model fungi. *Environ. Microbiol.* 18 5–20. 10.1111/1462-2920.13055 26373782 PMC4757429

[B19] Di Tommaso P. Moretti S. Xenarios I. Orobitg M. Montanyola A. Chang J.-M. (2011). T-Coffee: a web server for the multiple sequence alignment of protein and RNA sequences using structural information and homology extension. *Nucleic Acids Res.* 39 W13–W17. 10.1093/nar/gkr245 21558174 PMC3125728

[B20] Djamei A. Kahmann R. (2012). *Ustilago maydis*: dissecting the molecular interface between pathogen and plant. *PLoS Pathog.* 8:e1002955. 10.1371/journal.ppat.1002955 23133380 PMC3486881

[B21] Ernst O. P. Lodowski D. T. Elstner M. Hegemann P. Brown L. S. Kandori H. (2014). Microbial and animal rhodopsins: structures, functions, and molecular mechanisms. *Chem. Rev.* 114 126–163. 10.1021/cr4003769 24364740 PMC3979449

[B22] Estrada A. F. Avalos J. (2009). Regulation and targeted mutation of opsA, coding for the NOP-1 opsin orthologue in *Fusarium fujikuroi*. *J. Mol. Biol.* 387 59–73. 10.1016/j.jmb.2009.01.057 19361430

[B23] Estrada A. F. Brefort T. Mengel C. Díaz-Sánchez V. Alder A. Al-Babili S. (2009). *Ustilago maydis* accumulates β-carotene at levels determined by a retinal-forming carotenoid oxygenase. *Fungal Genet. Biol.* 46 803–813. 10.1016/j.fgb.2009.06.011 19584000

[B24] Fan Y. Solomon P. Oliver R. P. Brown L. S. (2011). Photochemical characterization of a novel fungal rhodopsin from *Phaeosphaeria nodorum*. *Biochim. Biophys. Acta* 1807 1457–1466. 10.1016/j.bbabio.2011.07.005 21791197

[B25] Feldbauer K. Schlegel J. Weissbecker J. Sauer F. Wood P. G. Bamberg E. (2016). Optochemokine tandem for light-control of intracellular Ca2+. *PLoS One* 11:e0165344. 10.1371/journal.pone.0165344 27768773 PMC5074463

[B26] Fernandes T. R. Segorbe D. Prusky D. Di Pietro A. (2017). How alkalinization drives fungal pathogenicity. *PLoS Pathog.* 13:e1006621. 10.1371/journal.ppat.1006621 29121119 PMC5679519

[B27] Feyder S. De Craene J.-O. Bär S. Bertazzi D. Friant S. (2015). Membrane trafficking in the yeast *Saccharomyces cerevisiae* model. *Int. J. Mol. Sci.* 16 1509–1525. 10.3390/ijms16011509 25584613 PMC4307317

[B28] Fischer R. Aguirre J. Herrera-Estrella A. Corrochano L. M. (2016). The complexity of fungal vision. *Microbiol. Spectr.* 4:FUNK-0020-2016. 10.1128/microbiolspec.FUNK-0020-2016 28087932

[B29] Freitag J. Lanver D. Böhmer C. Schink K. O. Bölker M. Sandrock B. (2011). Septation of infectious hyphae is critical for appressoria formation and virulence in the smut fungus *Ustilago maydis*. *PLoS Pathog.* 7:e1002044. 10.1371/journal.ppat.1002044 21625538 PMC3098242

[B30] Fuller K. K. Dunlap J. C. Loros J. J. (2016). Fungal light sensing at the bench and beyond. *Adv. Genet.* 96 1–51. 10.1016/bs.adgen.2016.08.002 27968729

[B31] Furuse M. Tamogami J. Hosaka T. Kikukawa T. Shinya N. Hato M. (2015). Structural basis for the slow photocycle and late proton release in *Acetabularia rhodopsin* I from the marine plant *Acetabularia acetabulum*. *Acta Crystallogr. Sect. D Biol. Crystallogr.* 71 2203–2216. 10.1107/S1399004715015722 26527138

[B32] Gabriel R. Kesselmeier J. Planck M. Box P. O. (1999). Apoplastic solute concentrations of organic acids and mineral nutrients in the leaves of several fagaceae. *Plant Cell Physiol.* 40 604–612. 10.1093/oxfordjournals.pcp.a029583

[B33] García-Martínez J. Brunk M. Avalos J. Terpitz U. (2015). The CarO rhodopsin of the fungus *Fusarium fujikuroi* is a light-driven proton pump that retards spore germination. *Sci. Rep.* 5:7798. 10.1038/srep07798 25589426 PMC4295100

[B34] Ghosh A. (2014). Small heat shock proteins (HSP12, HSP20 and HSP30) play a role in *Ustilago maydis* pathogenesis. *FEMS Microbiol. Lett.* 361 17–24. 10.1111/1574-6968.12605 25251081

[B35] Govorunova E. G. Koppel L. A. (2016). The road to optogenetics: microbial rhodopsins. *Biochemistry* 81 928–940. 10.1134/S0006297916090029 27682165

[B36] Govorunova E. G. Sineshchekov O. A. Spudich J. L. (2016). *Proteomonas sulcata* ACR1: a fast anion channelrhodopsin. *Photochem. Photobiol.* 92 257–263. 10.1111/php.12558 26686819 PMC4914479

[B37] Gustafsson M. G. L. Shao L. Carlton P. M. Wang C. J. R. Golubovskaya I. N. Cande W. Z. (2008). Three-dimensional resolution doubling in wide-field fluorescence microscopy by structured illumination. *Biophys. J.* 94 4957–4970. 10.1529/biophysj.107.120345 18326650 PMC2397368

[B38] He Q. Cheng P. Yang Y. Wang L. Gardner K. H. Liu Y. (2002). White Collar-1, a DNA binding transcription factor and a light sensor. *Science* 297 840–843. 10.1126/science.1072795 12098705

[B39] Idnurm A. Heitman J. (2005). Light controls growth and development via a conserved pathway in the fungal kingdom. *PLoS Biol.* 3:e95. 10.1371/journal.pbio.0030095 15760278 PMC1064852

[B40] Inoue K. Ono H. Abe-Yoshizumi R. Yoshizawa S. Ito H. Kogure K. (2013). A light-driven sodium ion pump in marine bacteria. *Nat. Commun.* 4:1678. 10.1038/Ncomms2689 23575682

[B41] Jung K.-H. (2007). The distinct signaling mechanisms of microbial sensory rhodopsins in Archaea, Eubacteria and Eukarya†. *Photochem. Photobiol.* 83 63–69. 10.1562/2006-03-20-ir-853 16968113

[B42] Kneen M. Farinas J. Li Y. Verkman A. S. (1998). Green fluorescent protein as a noninvasive intracellular pH indicator. *Biophys. J.* 74 1591–1599. 10.1016/S0006-3495(98)77870-1 9512054 PMC1299504

[B43] Lanver D. Müller A. N. Happel P. Schweizer G. Haas F. B. Franitza M. (2018). The biotrophic development of *Ustilago maydis* studied by RNA-seq analysis. *Plant Cell* 30 300–323. 10.1105/tpc.17.00764 29371439 PMC5868686

[B44] Leyser O. (2018). Auxin signaling. *Plant Physiol.* 176 465–479. 10.1104/pp.17.00765 28818861 PMC5761761

[B45] Li S. Hu P. C. Malmstadt N. (2011). Imaging molecular transport across lipid bilayers. *Biophys. J.* 101 700–708. 10.1016/j.bpj.2011.06.044 21806938 PMC3145269

[B46] Lorenzi R. Ceccarelli N. Lercari B. Gualtieri P. (1994). Identification of retinal in higher plants: is a rhodopsinlike protein the blue light receptor? *Phytochemistry* 36 599–600. 10.1016/S0031-9422(00)89781-2

[B47] Losi A. Polverini E. Quest B. Gärtner W. (2002). First evidence for phototropin-related blue-light receptors in prokaryotes. *Biophys. J.* 82 2627–2634. 10.1016/S0006-3495(02)75604-X 11964249 PMC1302051

[B48] Lyu X. Shen C. Fu Y. Xie J. Jiang D. Li G. (2015). The microbial opsin homolog Sop1 is involved in *Sclerotinia sclerotiorum* development and environmental stress response. *Front. Microbiol.* 6:1504. 10.3389/fmicb.2015.01504 26779159 PMC4703900

[B49] Mahlert M. Leveleki L. Hlubek A. Sandrock B. Bölker M. (2006). Rac1 and Cdc42 regulate hyphal growth and cytokinesis in the dimorphic fungus *Ustilago maydis*. *Mol. Microbiol.* 59 567–578. 10.1111/j.1365-2958.2005.04952.x 16390450

[B50] Mahyad B. Janfaza S. Hosseini E. S. (2015). Bio-nano hybrid materials based on bacteriorhodopsin: potential applications and future strategies. *Adv. Colloid Interface Sci.* 225 194–202. 10.1016/j.cis.2015.09.006 26506028

[B51] Matsuno-Yagi A. Mukohata Y. (1980). ATP synthesis linked to light-dependent proton uptake in a rad mutant strain of *Halobacterium* lacking bacteriorhodopsin. *Arch. Biochem. Biophys.* 199 297–303. 10.1016/0003-9861(80)90284-2 7356338

[B52] Moulton J. E. (1942). Extraction of auxin from maize, from smut tumors of maize, and from *Ustilago zeae*. *Bot. Gaz.* 103 725–739. 10.1086/335090

[B53] Nagel G. Ollig D. Fuhrmann M. Kateriya S. Mustl A. M. Bamberg E. (2002). Channelrhodopsin-1: a light-gated proton channel in green algae. *Science* 296 2395–2398. 10.1126/science.1072068 12089443

[B54] Nagel G. Szellas T. Huhn W. Kateriya S. Adeishvili N. Berthold P. (2003). Channelrhodopsin-2, a directly light-gated cation-selective membrane channel. *Proc. Natl. Acad. Sci. U.S.A.* 100 13940–13945. 10.1073/pnas.1936192100 14615590 PMC283525

[B55] Oesterhelt D. Stoeckenius W. (1971). Rhodopsin-like protein from the purple membrane of *Halobacterium halobium*. *Nature* 233 149–152. 10.1038/10.1038/NEWBIO233149A0 4940442

[B56] Oliva C. Cohen I. S. Mathias R. T. (1988). Calculation of time constants for intracellular diffusion in whole cell patch clamp configuration. *Biophys. J.* 54 791–799. 10.1016/S0006-3495(88)83017-0 3242629 PMC1330389

[B57] Olson D. K. Yoshizawa S. Boeuf D. Iwasaki W. DeLong E. F. (2018). Proteorhodopsin variability and distribution in the North Pacific Subtropical Gyre. *ISME J.* 12 1047–1060. 10.1038/s41396-018-0074-4 29476140 PMC5864233

[B58] Paula S. Tittor J. Oesterhelt D. (2001). Roles of cytoplasmic arginine and threonine in chloride transport by the bacteriorhodopsin mutant D85T. *Biophys. J.* 80 2386–2395. 10.1016/S0006-3495(01)76208-X 11325738 PMC1301427

[B59] Petersson S. V. Johansson A. I. Kowalczyk M. Makoveychuk A. Wang J. Y. Moritz T. (2009). An auxin gradient and maximum in the arabidopsis root apex shown by high-resolution cell-specific analysis of IAA distribution and synthesis. *Plant Cell* 21 1659–1668. 10.1105/tpc.109.066480 19491238 PMC2714926

[B60] Reineke G. Heinze B. Schirawski J. Buettner H. Kahmann R. Basse C. W. (2008). Indole-3-acetic acid (IAA) biosynthesis in the smut fungus *Ustilago maydis* and its relevance for increased IAA levels in infected tissue and host tumour formation. *Mol. Plant Pathol.* 9 339–355. 10.1111/j.1364-3703.2008.00470.x 18705875 PMC6640242

[B61] Savojardo C. Martelli P. L. Fariselli P. Profiti G. Casadio R. (2018). BUSCA: an integrative web server to predict subcellular localization of proteins. *Nucleic Acids Res.* 46 W459–W466. 10.1093/nar/gky320 29718411 PMC6031068

[B62] Schindelin J. Arganda-Carreras I. Frise E. Kaynig V. Longair M. Pietzsch T. (2012). Fiji: an open-source platform for biological-image analysis. *Nat. Methods* 9 676–682. 10.1038/nmeth.2019 22743772 PMC3855844

[B63] Schobert B. Lanyi J. K. (1982). Halorhodopsin is a light-driven chloride pump. *J. Biol. Chem.* 257 10306–10313. 7107607

[B64] Schulz B. Banuett F. Dahl M. Schlesinger R. Schäfer W. Martin T. (1990). The b alleles of *U. maydis*, whose combinations program pathogenic development, code for polypeptides containing a homeodomain-related motif. *Cell* 60 295–306. 10.1016/0092-8674(90)90744-Y 1967554

[B65] Shevchenko V. Mager T. Kovalev K. Polovinkin V. Alekseev A. Juettner J. (2017). Inward H + pump xenorhodopsin: mechanism and alternative optogenetic approach. *Sci. Adv.* 3:e1603197. 10.1126/sciadv.1603187 28948217 PMC5609834

[B66] Shoji J. Arioka M. Kitamoto K. (2006). Vacuolar membrane dynamics in the filamentous fungus *Aspergillus oryzae*. *Eukaryot. Cell* 5 411–421. 10.1128/EC.5.2.411-421.2006 16467481 PMC1405889

[B67] Smith H. Casal J. J. Jackson G. M. (1990). Reflection signals and the perception by phytochrome of the proximity of neighbouring vegetation. *Plant Cell Environ.* 13 73–78. 10.1111/j.1365-3040.1990.tb01301.x

[B68] Sosa-Morales M. E. Guevara-Lara F. Martínez-Juárez V. M. Paredes-López O. (1997). Production of indole-3-acetic acid by mutant strains of *Ustilago maydis* (maize smut / huitlacoche). *Appl. Microbiol. Biotechnol.* 48 726–729. 10.1007/s002530051123

[B69] Spudich E. N. Sundberg S. A. Manor D. Spudich J. L. (1986). Properties of a second sensory receptor protein in *Halobacterium halobium* phototaxis. *Proteins* 1 239–246. 10.1002/prot.340010306 3449857

[B70] Tsukuda T. Carleton S. Fotheringham S. Holloman W. K. (1988). Isolation and characterization of an autonomously replicating sequence from *Ustilago maydis*. *Mol. Cell. Biol.* 8 3703–3709. 10.1128/MCB.8.9.3703 2851726 PMC365426

[B71] Turian G. Hamilton R. H. (1960). Chemical detection of 3-indolylacetic acid in *Ustilago zeae* tumors. *Biochim. Biophys. Acta* 41 148–150. 10.1016/0006-3002(60)90381-4 13839903

[B72] Wang Z. Wang J. Li N. Li J. Trail F. Dunlap J. C. (2018). Light sensing by opsins and fungal ecology: NOP-1 modulates entry into sexual reproduction in response to environmental cues. *Mol. Ecol.* 27 216–232. 10.1111/mec.14425 29134709 PMC5797489

[B73] Waschuk S. A. Bezerra A. G. Shi L. Brown L. S. (2005). *Leptosphaeria* rhodopsin: bacteriorhodopsin-like proton pump from a eukaryote. *Proc. Natl. Acad. Sci. U.S.A.* 102 6879–6883. 10.1073/pnas.0409659102 15860584 PMC1100770

