# Supplementary material for: Opsin 1 and Opsin 2 of the Corn Smut Fungus Ustilago maydis Are Green Light-Driven Proton Pumps
Source: Front Microbiol. 2019 Apr 10;10:735. doi: 10.3389/fmicb.2019.00735 (PMC6467936; doi:10.3389/fmicb.2019.00735)
Supplement: Supplementary file 1 [file Data_Sheet_1.pdf]

Supplementary Material

Supplementary Table 1. Primers used in this study.

**Primersequence 5’ → 3’**	**Description (Name)**
ATATAATATTCGCCTCTCTCTAGGCAATCTACC	UMAG_03180 (wco1) LF fwd
ATATGGCCATCTAGGCCTTGAAGGGGCAATCACTAATTGC	UMAG_03180 (wco1) LF rev
ATATGGCCTGAGTGGCCAGCGCGTACCAAGCTCTCTCTCC	UMAG_03180 (wco1) RF fwd
ATATAATATTCTTATTACTTACTACCACAGTGC	UMAG_03180 (wco1) RF rev
ATATAATATTGAGCACGCTTCCGGTCGACGCG	UMAG_05732 (phy1) LF fwd
ATATGGCCATCTAGGCCGCGATCCGCCTAGACGTATAGC	UMAG_05732 (phy1) LF rev
ATATGGCCTGAGTGGCCTGTTGTCTGTGTTGAAGAATGG	UMAG_05732 (phy1) RF fwd
ATATAATATTGGTTCGAGCTTGGTTGCGATTCG	UMAG_05732 (phy1) RF rev
GTAACGCCAGGGTTTTCCCAGTCACGACGATTTAAATTTAGAGCAACCTCGTGGTGAAAGAGG	UMAG_02629 (ops1) LF fwd
CAATTGTCACGCCATGGTGGCCATCTAGGCCATCATTTTCCAGTCACCAACGCGAGAATTTTTTAC	UMAG_02629 (ops1) LF rev
GTGCGGCCGCATTAATAGGCCTGAGTGGCCGTTGAGAAGCTTCACGGATCAAGGAGGTGACACG	UMAG_02629 (ops1) RF fwd
GCGGATAACAATTTCACACAGGAAACAGCATTTAAATCACGAGTGTCGCACGAGGCAGTAGAAC	UMAG_02629 (ops1) RF rev
GTAACGCCAGGGTTTTCCCAGTCACGACGAATATTCTCGTGTCTTGCTCGTTGTTAATAAAC	UMAG_00371 (ops2) LF fwd
CAATTGTCACGCCATGGTGGCCATCTAGGCCGTTGAGTCAGAGCAGGCAAAGCGCGAAGC	UMAG_00371 (ops2) LF rev
GTGCGGCCGCATTAATAGGCCTGAGTGGCCACCACTCGATTACCGCGTTGCACTCGCCCCTTG	UMAG_00371 (ops2) RF fwd
GCGGATAACAATTTCACACAGGAAACAGCAATATTATCGATCTGGAAGCCAAGTTTGCGCCG	UMAG_00371 (ops2) RF rev
ATGGTACCATGAACGTCGTATCCGAGCTGC	UMAG_02629 fwd for p1747
ATGGATCCCTGGGTAACGGTGTGCATTTGG	UMAG_02629 rev for p1747
ATGGATCCATGAACTCGTTCTACTACGACAGCGTC	UMAG_00371 fwd for p1742
TAATCCCGGGGGCGTCTTCAGCGCGGGGTTC	UMAG_00371 rev for p1742
GAACTTCAAGCTCTCGCAC	UMAG_03726 (cpr1) qRT-PCR fwd
TCTCGATAGCCTTGACGATG	UMAG_03726 (cpr1) qRT-PCR rev
ACTCTTCGCTCGTTCTCACC	UMAG_02629 (ops1) qRT-PCR fwd
CCTCGTAGTCTGAAAACTTGCC	UMAG_02629 (ops1) qRT-PCR rev
TTTGCTCGATACATTGACTGG	UMAG_00371 (ops2) qRT-PCR fwd
GGATGAACACCAGCAATCAC	UMAG_00371 (ops2) qRT-PCR rev
ACTAAGCTTATGAACGTCGTATCCGAGCTGCTG	ops1 fwd for pcDNA^{TM5}/FRT/TO$^{©}$
TGGCGGCCGCCTGGGTAACGGTGTGCATAAG	ops1 rev for pcDNA^{TM5}/FRT/TO$^{©}$
ACTAAGCTTATGAACTCGTTCTACTACGACAGC	ops2 fwd for pcDNA^{TM5}/FRT/TO$^{©}$
TGGCGGCCGCGGCGTCTTCAGCGCGGGGTTC	ops2 rev for pcDNA^{TM5}/FRT/TO$^{©}$
ACTAAGCTTATGTTCACAAACATCTTGCTCAAACGC	ops3 fwd for pcDNA^{TM5}/FRT/TO$^{©}$
TGGCGGCCGCGGTAACAAGAGTGTGTGGAACG	ops3 rev for pcDNA^{TM5}/FRT/TO$^{©}$
CCATCTTTTTCGATTGGGTCATGATCATCACC	187t_SDM_Primer_L149W_UmOps1_fwd
GGTGATGATCATGACCCAATCGAAAAAGATGG	188t_SDM_Primer_L149W_UmOps1_rev
CCTGCTCCTCGACATCCTGCTTG	205t_UmOps1_E129D_fwd
CAAGCAGGATGTCGAGGAGCAGG	206t_UmOps1_E129D_rev
GGTCTCGCCGAGGGTAGCAACACAATTTCG	207t_UmOps1_D225E_fwd
CGAAATTGTGTTGCTACCCTCGGCGAGACC	208t_UmOps1_D225E_rev

Supplementary Table 2. Overview of various *U. maydis* strains expressing eGFP-tagged UmOps1 or UmOps2.

UmOps1 (UMAG_02629)			
Name	Description	Resistance	Microscopic evaluation
FB1 ΔUmOps1 *pcrg*-UmOps1::eGFP #1	genomic deletion of UMAG_02629 + single genomic insertion of pcrg-UMAG_02629::eGFP into *ip*-locus (pCRG-GFP-MXN)	Hyg, Cbx	See Figure 5.
FB1 *pcrg*-UmOps1::eGFP #A	multiple genomic insertions of pcrg-UMAG_02629::eGFP into *ip*-locus (pCRG-GFP-MXN)	Cbx	Localization in plasma membrane; in addition accumulation in the endoplasmic reticulum while the promoter is active.
UmOps2 (UMAG_00371)			
FB1 ΔUmops2 *petef*-UmOps2::eGFP #1	genomic deletion of UMAG_00371 + multiple genomic insertions of *petef-UMAG_00371::eGFP* into *ip*-locus (pETEF-GFP-MXN)	Hyg, Cbx	Localization in vacuolar membrane; in addition accumulation in the endoplasmic reticulum.
FB1 ΔUmOps2 *petef*-UmOps2::eGFP #9	genomic deletion of UMAG_00371 + single genomic insertion of *petef-UMAG_00371::eGFP* into *ip*-locus (pETEF-GFP-MXN)	Hyg, Cbx	See Figure 5.
FB1 *pcrg*-UmOps2::eGFP #1	multiple genomic insertions of *pcrg-UMAG_00371::eGFP* into *ip*-locus (pCRG-GFP-MXN)	Cbx	Localization in vacuolar membrane; in addition accumulation in the endoplasmic reticulum.
FB1 *pcrg*-UmOps2::eGFP #7	multiple genomic insertions of *pcrg-UMAG_00371::eGFP* into *ip*-locus (pCRG-GFP-MXN)	Cbx	See supplementary Figure 8. Localization in vacuolar membrane; in addition accumulation in the endoplasmic reticulum when the promoter is active.
FB1 ΔUmOps2 *pcrg*-UmOps2::eGFP #B	genomic deletion of UMAG_00371 + multiple genomic insertions of *pcrg-UMAG_00371::eGFP* into *ip*-locus (pCRG-GFP-MXN)	Hyg, Cbx	Localization in vacuolar membrane; in addition accumulation in the endoplasmic reticulum also several hours after inactivation of the promoter.

```
Hal.sal.BR      MLEL------ ---------- ---------- ----LPTAVE GVSQAQITGR PEWIWLALGT ALMGLGTLYF
Fus.fuj.CarO    MAD-----HL YAR-KN---- ------DAL- --NVNPDIVN GQRSDINITV RGSDWYWAVC AVMTVSTFAF
Neu.cra.Nop1    MIHPEQVADM LRPTTST--- ---TSSHVP- --GPVPTVVP TPTEYQTLGE TGHRTLWVTF ALMVLSSGIF
Lep.mac.ops1    MIVD-QFEEV LMKTSQLFPL PTATQSAQPT HVAPVPTVLP DTPIYETVGD SGSKTLWVVF VLMLIASAAF
Ust.may.ops1    MNVV---SEL LKRAGN---- ------MAL- --SSNPT--- --VADIDITT PGSDWLWAVF SVMAATGLGT
Ust.may.ops2    MNSF-YYDSV LDA------- ----FKKPE- --VGIPPIPP HLEHPIVSTQ WGHTYLWVVF FIMAGFSLLF
Ust.may.ops3    MFT----NIL LKRAGN---- ------RAL- --DVNPP--- --NADIHLGR WGSDWGWVVF CVMAASTLGL
Clustal Cons    *                                     *                            :*

Hal.sal.BR      LVKGMGVSDP DAKKFYAITT LVPAIAFTMY LSMLLGYGLT MVPFG----- ---------G EQNPIYWARY
Fus.fuj.CarO    LGLGMRKPR- TDRIFHYITA GITMIASIAY FTMASNLGWT PIAVEF-QRS NHR--VAG-- IYREIFYARY
Neu.cra.Nop1    ALLSWNVPT- SKRLFHVITT LITVVASLSY FAMATGHATT FNCDTAWDHH KH-VPDTSHQ VCRQVFWGRY
Lep.mac.ops1    TALSWKIPV- NRRLYHVITT IITLTAALSY FAMATGHGVA LNKIVIRTQH DH-VPDTYET VYRQVYYARY
Ust.may.ops1    MVWSLKVSR- GERAFHYLSA AILATASVAY FSMASDLGAT PVRVEF-TNY GPN-EVNGLR PTRSIWYVRY
Ust.may.ops2    FISSQRASY- RYRLMHTTTF FITAIAALSY FAMATGVGKT LSTIG----- ------GGHH APREFYFARY
Ust.may.ops3    LVWTFILPR- KRRTFHYLLT AVLAISTISW YSQASDLGAT PITTQF-LHN SQRGGGYGGY PTRQIWYSRY
Clustal Cons           .     :  :      :   :   :  :   . . :                         . .:: **
                                                                                          82 83

Hal.sal.BR      ADWLFTTPLL LLDLALLVDA DQGTILALVG ADGIMIGTGL VGALTKV--Y SYRFVWWAIS TAAMLYILYV
Fus.fuj.CarO    IDWFLTTPLL LTDLLLTAGM PWPTVLWVIL VDWVMIVTGL VGALVK---S SYKWGYFAFG CAALAYIVYV
Neu.cra.Nop1    VDWALTTPLL LLELCLLAGV DGAHTLMAIV ADVIMVLCGL FAALGEGGNT AQKWGWYTIG CFSYLFVIWH
Lep.mac.ops1    IDWAITTPLL LLDLGLLAGM SGAHIFMAIV ADLIMVLTGL FAAFGSE-GT PQKWGWYTIA CIAYIFVVWH
Ust.may.ops1    IDWTVTTPLL LLEILLVSGL PLSTVFITIF FDLVMIITGL IGSLVE---S TYKWGYYTMG CVAMFYVFWV
Ust.may.ops2    IDWALTTPLL LFDLTLLAGL PVAEIIVLVL ADEVMIVTGV IAGVHPT--K TGKWGFFTFS CIAFAWVLLQ
Ust.may.ops3    VDWTLTTTLL LLSLLLITGL PLSIIFITLF FNILMIVCGL LGALTR---T RYKWGYFAFA CAALGYVLYH
Clustal Cons     ** .**.** * .: *  .       :  :   : :*:  *: ....         :: ::::.   :  ::.
                 85 86 89  93  96                  116

Hal.sal.BR      LFFGFTSKAE SMRPEVASTF KVLRNVTVVL WSAYPVVWLI GSEGAGIVPL NIETLLFMVL DVSAKVGFGL
Fus.fuj.CarO    LAWEARLHAK HVGPDVGRTF VMCGSLTAVV WILYPIAWGV -CEGGNLIAP DSEAVFYGIL DLIAKPVFGA
Neu.cra.Nop1    VALHGSRTVT AKGRGVSRLF TGLAVFALLL WTAYPIIWGI -AGGARRTNV DTEILIYTVL DLLAKPVFGF
Lep.mac.ops1    LVLNGGANAR VKGEKLRSFF VAIGAYTLIL WTAYPIVWGL -ADGARKIGV DGEIIAYAVL DVLAKGVFGA
Ust.may.ops1    IYGPGLKSAS HLGADFKKAY LYSSLVLTIL WTLYPVAWGL -ADGSNTISP NAEMIFYGVL DLLAKPVFAL
Ust.may.ops2    LVTSGRSTAF LRSPKVGGLY NQVSLALVVV WTAYPVAFAL -CEGTGKLNP DAEILFFAIL DVIAKPLWGA
Ust.may.ops3    VFASGLRSSR RLGSRFGRAF LAASLLLAII WPMYPICWGL -SEGGNVIGV SAEFLWYGLL DIFSKPIEAF
Clustal Cons    :               .   :          :: *  **: : :  . *       . * : : :* *: :*  :.
                                            178  182  185 189   194       204      212 216 219

Hal.sal.BR      ILLRSRAIF- ----GEAEAP EP-SA----- ---------- -GDG------ ---------A AATS------
Fus.fuj.CarO    LLLWGHRNI- ----DPARLG LRIRDIDERI FPDGPN---- ---------- NKVASG-HGA RNDTATAS-G
Neu.cra.Nop1    WLLLSHRAM- ----PETNID LP-GYW---- ---------- -SHG------ ---------L ATEGR-----
Lep.mac.ops1    WLLVTHANL- ----RESDVE LN-GFW---- ---------- -ANG------ ---------L NREGA-----
Ust.may.ops1    FHLWSLRRC- ----NYSSLH LKSGKFSD-- YEDLSGNHYR SMEGGKASEA GVTGAGVNGV -ATGVNTTGA
Ust.may.ops2    WLLLATPDEG HVLVPESLCA PA-GSP---- ---------- -ASG------ ------GYGA ISQEP-----
Ust.may.ops3    LFLGMLKKC- ----DYGALR LRSGKRSE-- -EDSD----- ---------V HEPR------ -RENMEKD-H
Clustal Cons      *

Hal.sal.BR      --------D- ---------- -
Fus.fuj.CarO    SNVN--PNA- ---------- -
Neu.cra.Nop1    IRIGEE--D- ---------- -
Lep.mac.ops1    IRIGEDDGA- ---------- -
Ust.may.ops1    DVTGANVLHP APPTQMHTVT Q
Ust.may.ops2    --RAED--A- ---------- -
Ust.may.ops3    ERIG------ ---------- -
Clustal Cons
```

Supplementary Figure 1. Alignment of the amino acid sequences of selected microbial rhodopsins. The alignment includes BR (*Halobacterium salinarum*, AAA72504), CarO (*Fusarium fujikuroi*, CAD97459), Nop1 (*Neurospora crassa*, XP_959421), LR (*Leptosphaeria maculans*, AAG01180), and the three *Ustilago maydis* rhodopsins UmOps1 (UMAG_02629), UmOps2 (UMAG_00371), and UmOps3 (UMAG_04125). Functionally and structurally important residues are highlighted by colored boxes: red (proton donor/-acceptor), green (ion transport), blue (Schiff base), orange (structural importance), grey (putative interaction site in CarO-like rhodopsins). Numbers below the alignment represent the respective position of the BR sequence.

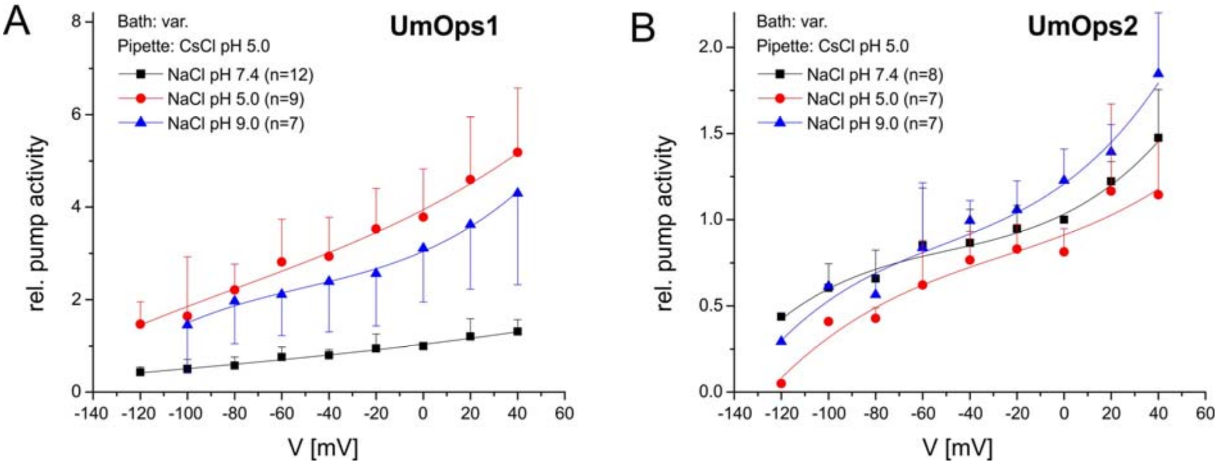

Supplementary Figure 2. Pump activity of UmOps1 (A) and UmOps2 (B) in absence of intracellular sodium. I-V-plots of the pump current in CsCl-based pipette solution at different membrane potentials and extracellular pH values as indicated. Measurements in TEA-Cl were similar but did not yield satisfying signal-to-noise ratios. In both conditions, the pump characteristics of both rhodopsins were hardly influenced by the absence or presence of sodium ions. For comparison see Figure 3B and 3C.

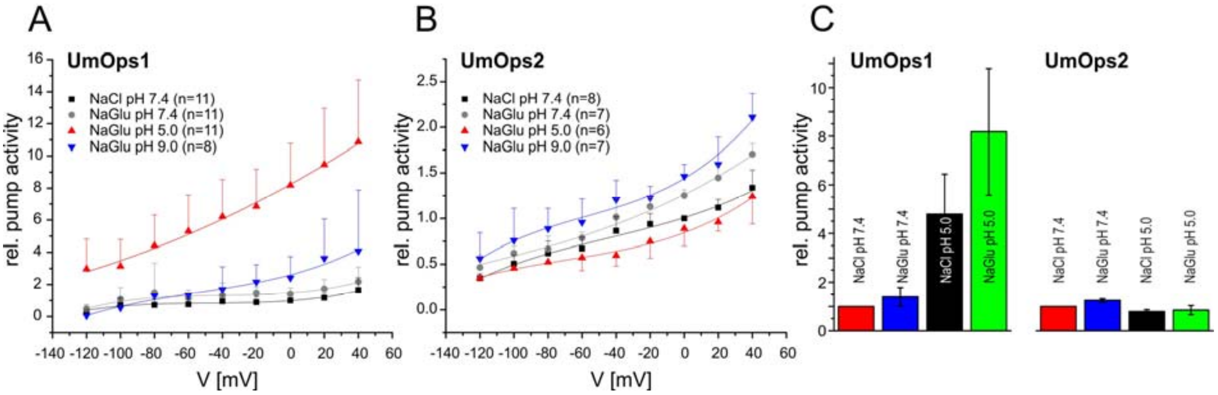

Supplementary Figure 3. Pump activity of UmOps1 (A) and UmOps2 (B) in absence of extracellular chloride. I-V-plots of the pump current in NaCl pH 7.4 and sodium gluconate-based bath solutions at different membrane potentials and extracellular pH values as indicated. In general, the pump characteristics of UmOps1 and UmOps2 were similar in gluconate- as in chloride-based (Fig. 3 B, C) solutions but slightly enhanced. **C.** Relative pump activity at 0 mV at different extracellular conditions. The presence of sodium gluconate in general leads to a slight enhancement of the pump activity when compared with NaCl. The supporting effect is very pronounced in UmOps1 at pH 5.0.

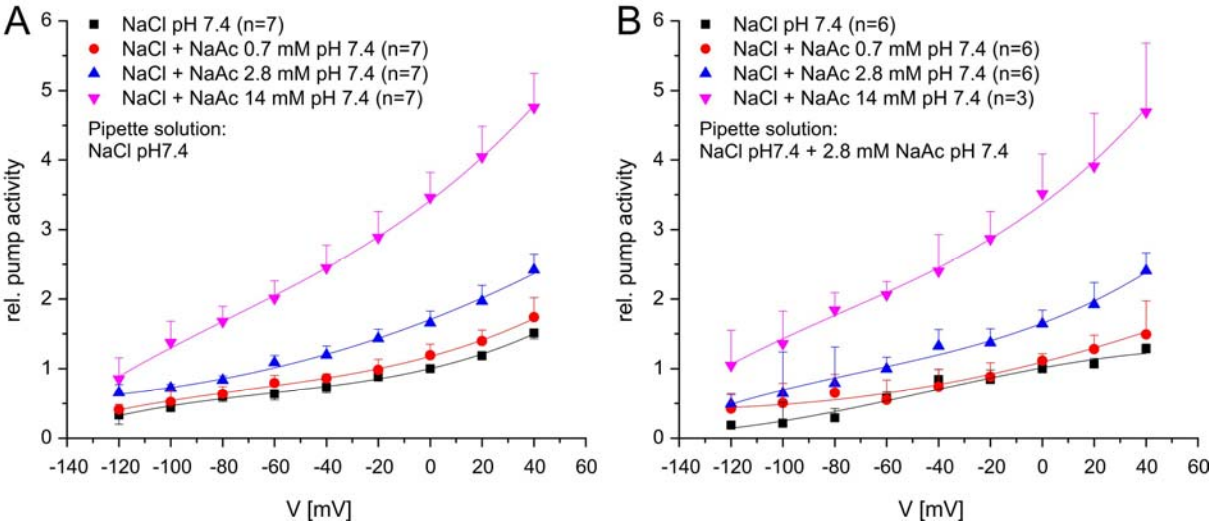

Supplementary Figure 4. Supporting effect of sodium acetate on UmOps1 pump activity at different concentrations and intra- and extracellular pH 7.4. The pipette solution was either untreated (**A**) or supplemented with 2.8 mM sodium acetate (**B**). Though the pump activity of UmOps1 is much lower in neutral environment than in pH 5.0 (see Fig. 4C), the supporting effect of sodium acetate is still present. The behavior is essentially the same when sodium acetate is absent or present in the pipette solution, suggesting that the interaction of WOAs and UmOps1 occurs in the extracellular part of the protein.

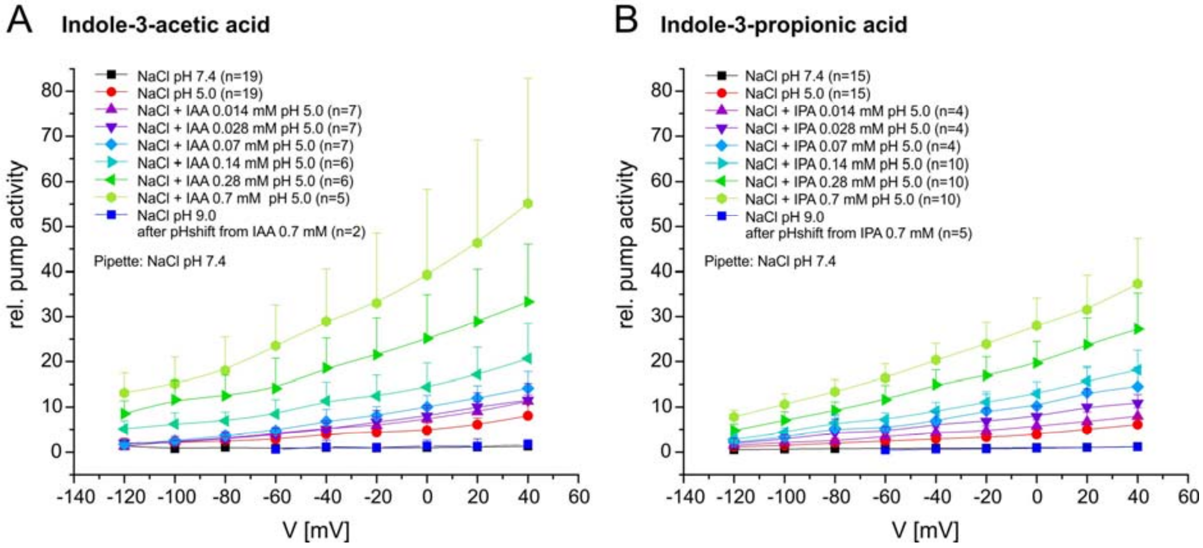

Supplementary Figure 5. Increase of pump activity of UmOps1 in presence of Indole-3-acetic acid (IAA, A) and Indole-3-propionic acid (IPA, B). I-V-plots of the pump currents in extracellular NaCl pH 7.4 and NaCl pH 5.0 with different concentrations of IAA / IPA at different membrane potentials as indicated.

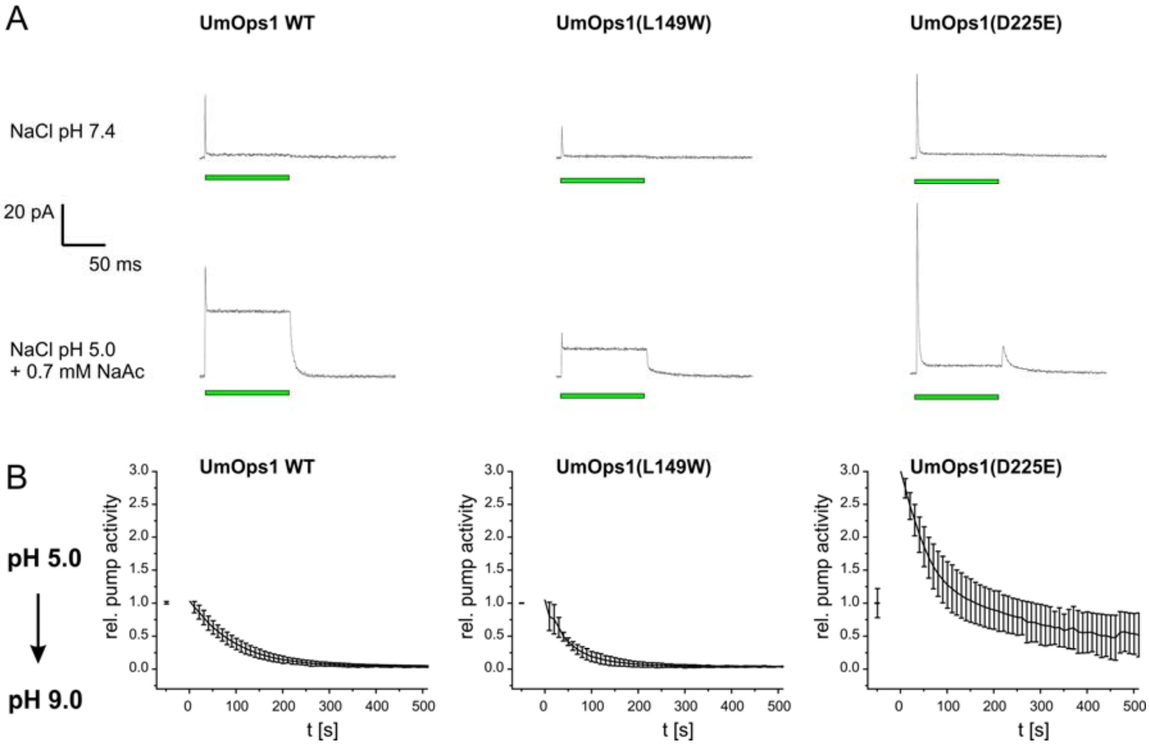

Supplementary Figure 6. Whole cell patch-clamp analysis of UmOps1 mutants L149W and D225E in comparison with WT. A. Typical traces showing the light-induced charge transfer by UmOps1 as indicated by the green bars. The pump intensity of UmOps1 WT and L149W strongly increased in the presence of WOAs at external pH 5.0 (lower raw) compared to NaCl pH 7.4 (upper raw), this behavior is less pronounced in D225E, which also exhibits a transient increase in pump activity after illumination indicating an electrogenic dark reaction. **B**. Relaxation of the UmOps1 pump activity after replacement of NaCl pH 5.0 + 7 mM sodium acetate by NaCl pH 9.0. Every 10 seconds the pump current was recorded. Using an exponential decay function, time constants of 89.2 s (WT), 56.5 s (L149W), and 89.7 s (D225E) were obtained. The D225E mutant exhibits a transient increased pump activity in pH 9.0, similar as described for CarO from *F. fujikuroi* (Adam et al., 2018).

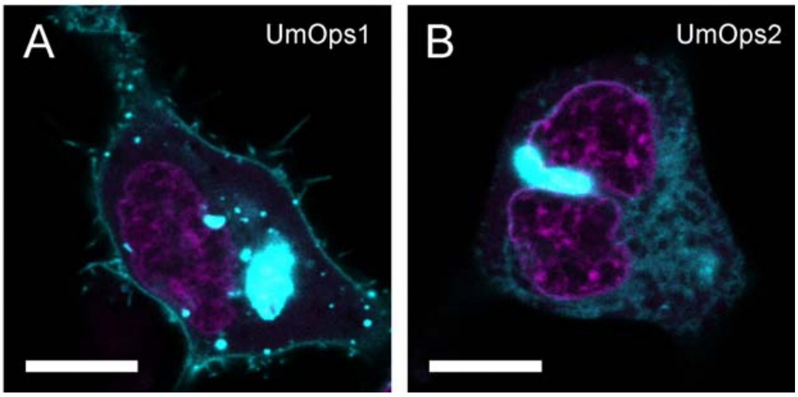

Supplementary Figure 7: Heterologous expression of eYFP-tagged UmOps1 (A) and UmOps2 (B) in HEK293 cells. Confocal laser scanning micrographs show the location of the respective rhodopsin (cyan) and the nucleus stained with SYTO® 59 (magenta). In accordance with our observations in sporidia (Fig. 5, main part), we found distinct eYFP mediated fluorescence in the plasma membrane of HEK293 UmOps1::eYFP but mainly in endomembranes in HEK293 UmOps2::eYFP cells. The low amount of UmOps2 in the plasma membrane is in accordance with small pump currents of this rhodopsin. Scale bars represent 10 µm.

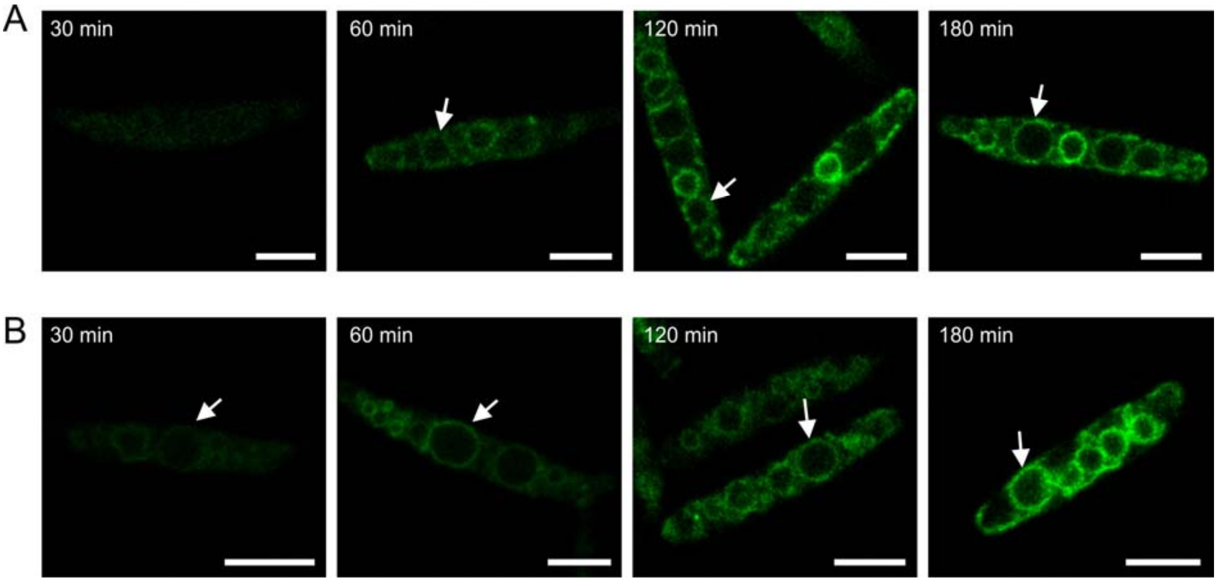

Supplementary Figure 8: CLSM analysis of the localization of UmOps2::eGFP in *U. maydis* sporidia in dependence of the expression time. The strain FB1 *pcrg*-UmOps2::eGFP with an arabinose-inducible promoter was used for this analysis. Protein localization after promoter on-times between 30 min and 180 min were analyzed as indicated (time of exposure to arabinose). The samples were fixed with 1% formaldehyde either 25 min (A) or 195 min (B) after the removal of arabinose allowing to trace the protein expression after stop of transcription. Note that the UmOps2::eGFP protein was never observed in the plasma membrane but only found in vacuolar membranes or after longer expression times also in the ER. All images were obtained using the same parameters for illumination and recording (4% laser power (488 nm), photomultiplier: 700). White bars represent 5 µm. Arrows highlight fluorescence in the vacuolar membranes.
